# Improved pharmacokinetics of HIV-neutralizing VRC01-class antibodies achieved by reduction of net positive charge on variable domain

**DOI:** 10.1080/19420862.2023.2223350

**Published:** 2023-06-21

**Authors:** Young D. Kwon, Amarendra Pegu, Eun Sung Yang, Baoshan Zhang, Michael F. Bender, Mangaiarkarasi Asokan, Qingbo Liu, Krisha McKee, Bob C. Lin, Tracy Liu, Mark K. Louder, Reda Rawi, Mateo Reveiz, Andrew J. Schaub, Chen-Hsiang Shen, Nicole A. Doria-Rose, Paolo Lusso, John R. Mascola, Peter D. Kwong

**Affiliations:** a Vaccine Research Center, National Institute of Allergy and Infectious Diseases, National Institutes of Health, Bethesda, MD, USA; b Laboratory of Immunoregulation, National Institute of Allergy and Infectious Diseases, NIH, Bethesda, MD, USA

**Keywords:** Antibody-mediated prevention, net positive charge reduction, pharmacokinetics, serum half-life, surface charge, variable region, VRC01-class antibody

## Abstract

The amino-acid composition of the immunoglobulin variable region has been observed to impact antibody pharmacokinetics (PK). Here, we sought to improve the PK of the broad HIV−1-neutralizing VRC01-class antibodies, VRC07-523LS and N6LS, by reducing the net positive charge in their variable domains. We used a structure-guided approach to generate a panel of antibody variants incorporating select Arg or Lys substituted to Asp, Gln, Glu, or Ser. The engineered variants exhibited reduced affinity to heparin, reduced polyreactivity, and improved PK in human FcRn-transgenic mice. One variant, VRC07-523LS.v34, with three charge substitutions, had an observed *in vivo* half-life and an estimated human half-life of 10.8 and 60 days, respectively (versus 5.4 and 38 days for VRC07-523LS) and retained functionality, neutralizing 92% of a 208-strain panel at a geometric mean IC_80_ <1 µg/mL. Another variant, N6LS.C49, with two charge substitutions, had an observed *in vivo* half-life and an estimated human half-life of 14.5 and 80 days (versus 9.0 and 44 days for N6LS) and neutralized ~80% of 208 strains at a geometric mean IC_80_ <1 µg/mL. Since Arg and Lys residues are prevalent in human antibodies, we propose substitution of select Arg or Lys with Asp, Gln, Glu, or Ser in the framework region as a general means to improve PK of therapeutic antibodies.

## Introduction

Therapeutic antibodies are widely used to treat a variety of diseases, including various cancers, autoimmune diseases, cardiovascular diseases, and infectious diseases, through their ability to recognize diverse targets with high specificity and relative safety.^1,2^ Passive transfer of highly potent and broadly reactive HIV−1 neutralizing antibodies also holds promise to prevent HIV−1 infection,^3–5^ as an alternative to a vaccine. Antibody-mediated prevention, however, requires repeated infusions of antibody doses and may be costly, prompting the development of antibodies with prolonged *in vivo* half-lives to make this approach more feasible and more affordable. Approaches to extend the *in vivo* half-life of antibodies have focused on enhancing pH-dependent interactions between the crystallizable fragment (Fc) of the antibody and the neonatal Fc receptor (FcRn), and include LS, YTE, and DHS mutations that yield substantially improved serum half-lives.^6–12^ However, these mutations have not been effective for all antibodies. Moreover, antibodies with identical Fc domains can have different levels of improvement in their half-lives and clearance rates, indicating that the antigen-binding fragment (Fab) also contributes to antibody homeostasis.^13–17^ Indeed, it has been suggested that variable domain charge can influence pharmacokinetics (PK)^16,18–21^ and it has been shown that off-target bindings between the Fab regions and vesicular cell membranes mediated by hydrophobic or electrostatic interactions are associated with increased antibody absorption by endocytosis and fast clearance rates.^22–24^ It has also been reported that antibody-antigen binding alters the dynamics of Fc-FcRn interactions to differentially affect the *in vivo* half-lives and clearance rates of antibodies.^25^ Other factors associated with the serum half-lives and clearance rates involved physical characteristics of antibodies, such as their solubility, thermal stability, and polyreactivity. Improved potency and breadth of antibodies by engineering or library-based screenings are often accompanied by enhanced polyreactivity, resulting in a fast clearance rate and reduced serum half-life.^26–29^

VRC01-class antibodies neutralize over 90% of HIV−1 strains,^30^ with improved versions such as VRC07–523^27^ and N6^31^ having even higher levels of neutralizing potency and breadth. Some attempts to improve further, such as through the incorporation of a tryptophan as residue 54,^32^ however, have led to increased polyreactivity and reduced *in vivo* PK.^27,32^ As VRC01-class antibodies have been suggested as prophylactics for their ability to prevent HIV−1 infection when administered prophylactically,^5^ maintenance, or improvement of their PK is critical to enable preservation of suitable antibody levels for a 6- or 12-month dosing interval.

Previously, we reported that insertion of antibody VRC03 framework region 3 (03FR3) loop to VRC01-class antibodies improved potency, reduced polyreactivity, and improved PK.^32^ However, the mechanistic basis for the reduced polyreactivity and improved PK was unclear. In this study, we investigated the basis for reduced polyreactivity and increased half-lives of VRC01-class antibodies and found that four aspartates introduced by 03FR3 loop insertion were responsible for reduced polyreactivity. Based on this finding, we hypothesized that reducing net positive charge of the variable domain, in general, could serve as a means to improve antibody half-life and sought to improve the half-lives of VRC01-class antibodies, VRC07-523LS and N6LS, which are among the best-in-class, in terms of neutralization potency and breadth. We generated a panel of antibody variants with select Arg or Lys mutated to Asp, Gln, Glu, or Ser, and assessed polyreactivity, neutralizing potency, affinity to heparin, and *in vivo* half-lives in human FcRn transgenic mice. Our findings reveal reduction in net positive charge of an antibody variable domain as a means to improve its serum PK, while maintaining function: high potency and breadth of HIV−1 neutralization.

## Results

### Basis of reduced polyreactivity by VRC03 framework region 3 loop insertion

**Figure 1.**
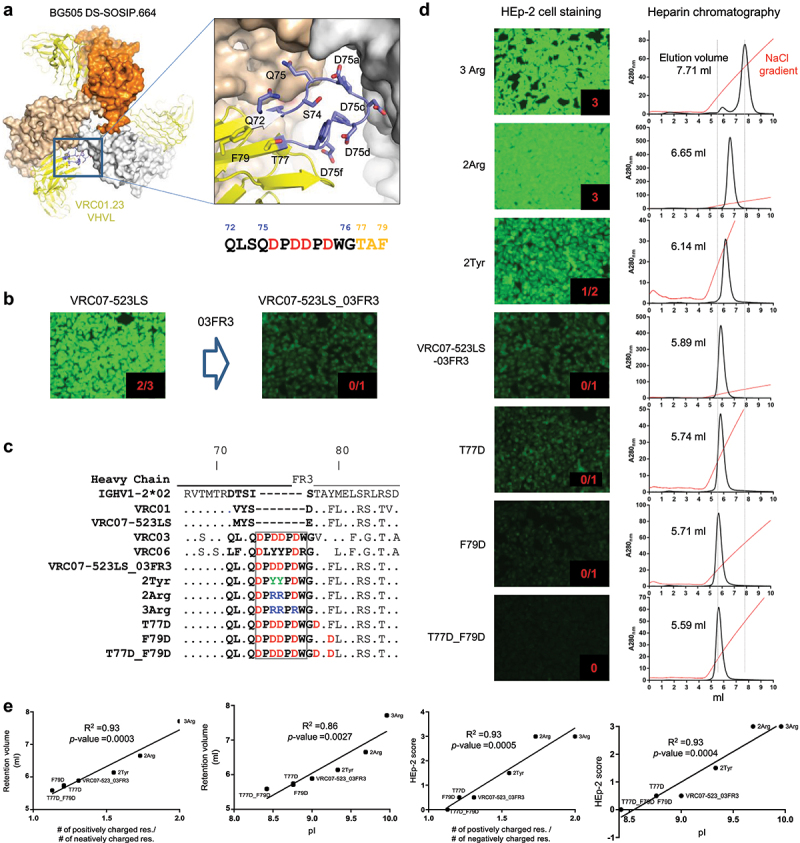
VRC07–523-03FR3 variants with reduced net positive charge showed reduced affinity to heparin and reduced polyreactivity. (a) Closeup view of 03FR3 loop insertion into VRC01.23LS heavy chain. Four Asp’s were highlighted in red. (b) Insertion of 03FR3 loop into VRC07-523LS reduced HEp−2 cell binding. Antibodies scored greater than 1 at 25 µg/ml were considered polyreactive. (c) Sequence alignments of VRC07-523LS variants (d) Polyreactivity of VRC07-523LS variants assessed in HEp−2 cell bindings (left panel). Chromatograms of VRC07-523LS variants on heparin affinity chromatography (right panel). Redlines represent the NaCl gradient. Retention volumes of VRC07-523LS variants on heparin chromatography correlated with polyreactivity. (e) Correlations of heparin retention volume vs. net positive charge, HEp−2 cell bindings vs. isoelectric point (pI), HEp−2 cell bindings vs. net positive charge of VRC07-523LS variants.

Previously, we reported that 03FR3 loop insertion to VRC01-class antibodies enables the antibodies to contact a second binding site, the CD4 binding site−2 (CD4BS–2) on a neighboring Env protomer, which is also recognized by CD4 and enhances their neutralizing potency against viruses tested.^33^ The insertion also reduces polyreactivity against HEp−2 cells. The basis for the reduced polyreactivity, however, was unclear. To understand the basis for the reduced polyreactivity, we focused on the four aspartic acid residues in the seven-residue insert (Figure 1a,b), and generated a panel of VRC07-523LS_03FR3 variants composed of two distinct groups: one group with more positively charged variants and the other with less positively charged variants than VRC07-523LS_03FR3 (Figure 1c). The former group included: i) VRC07-523LS_03FR3_2Tyr where the Asp75c and Asp75d were replaced with Tyr, the counterpart of VRC06; ii) VRC07-523LS_03FR3_2Arg where Asp75c and Asp75d were replaced with Arg; and iii) VRC07-523LS_03FR3_3Arg where Asp75c, Asp75d, and Asp75f were replaced with Arg. The latter group included: iv) VRC07-523LS_03FR3_T77D where Thr77 was replaced with Asp; v) VRC07-523LS_03FR3_F79D, where Phe79 was replaced with Asp; and vi) VRC07-523LS_03FR3_T77D_F79D where Thr77 and Phe79 were replaced with Asp.

Variants i)-iii) were expected to exhibit increased polyreactivity while variants iv)-vi) were expected to show further reduced polyreactivity than VRC07-523LS_03FR3 (Figure 1c). HEp−2 cell-binding assays on these variants showed what we anticipated. Variants i)-iii) comprising more positively charged mutations than VRC07-523LS_03FR3 showed increased polyreactivity in the rank order of 3Arg >2Arg >2Tyr, while variants iv)-vi) all showed further reduced polyreactivity and reduced affinity to heparin, demonstrating that the reduced net positive charge due to 03FR3 loop insertion contributes to the reduced polyreactivity (Figure 1d). Affinity to heparin significantly correlated with polyreactivity assessed in the HEp−2 cell-binding assay (Figure 1d). Thus, reduction in net positive charge either by replacing Arg to amino acids with polar or aromatic side chains or by introducing more Asp was directly associated with reduced polyreactivity and affinity to heparin (Figure 1d,e).

### Workflow to improve the PK of HIV−1 antibodies by reducing net positive charge in the variable domain

**Figure 2.**
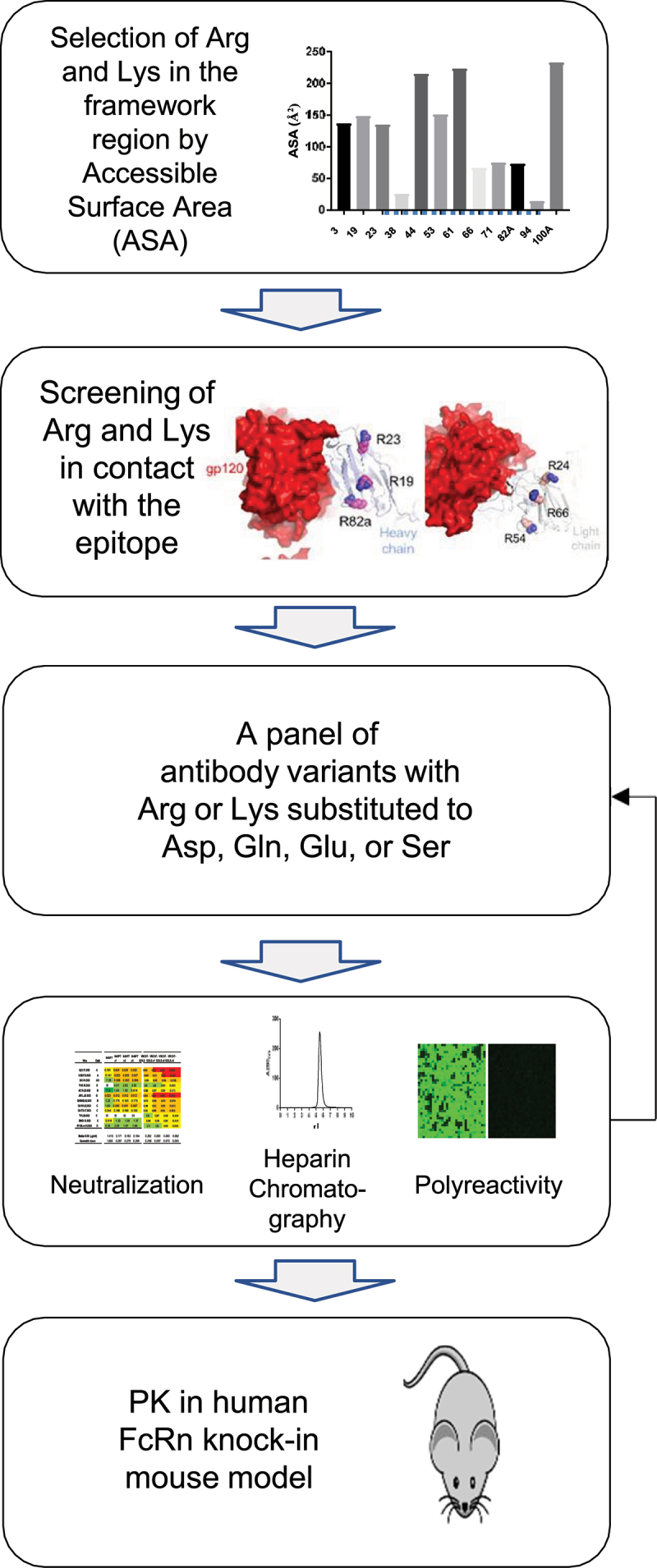
Workflow for identifying antibody variants with improved pharmacokinetics by surface charge alteration. (1) Selection of Arg or Lys residues in the framework region based on accessible surface areas. (2) Screening the residues that contact with the epitope or within 5Å from the epitope. (3) Generation of antibody variants.Selection of candidate variants for PK assessment based on neutralizing potency and breadth, polyreactivity, and retention volumes on heparin affinity chromatography. (5) Assessment of pharmacokinetics in human FcRn knock-in mouse model.

We sought to explore whether we could further improve the PK of VRC07-523LS and N6LS variants engrafted with the 03FR3 loop by substituting select Arg or Lys to Asp, Gln, Glu, or Ser in the FR. To select Arg and Lys residues for substitution, we first calculated the accessible surface area (ASA) of all Arg and Lys residues in the variable domain, and eliminated those with low ASA. The remaining Arg and Lys residues were assessed for those that made direct contact with epitopes, resided within 5 Å of an epitope, or made contacts with a neighboring protomer and removed them from further consideration (Figure 2). The final selected Arg and Lys residues in heavy and light chains were then substituted with Asp, Gln, Glu, or Ser, and variants incorporating these single mutations were evaluated for their neutralizing potency in a representative 12-strain panel. They were also assessed for their affinity to heparin. Single mutations that reduced affinity to heparin and retained or reduced potency by less than approximately 2-fold were selected and used to generate a panel of variants composed of single mutation combinations in heavy and light chains. Variants with the best neutralization potency, affinity to heparin, and polyreactivity were selected and assessed for PK in human FcRn knock-in mice (Figure 2).

### *A panel of VRC07-523LS variants with Arg or Lys to Asp, Gln, Glu, or Ser substitutions in the variable domain identified variants with improved* in vivo *half-life*

#### First round - screening of variants incorporating Arg to Asp substitutions selected based on ASA and a VRC07-bound gp120 structure

**Figure 3.**
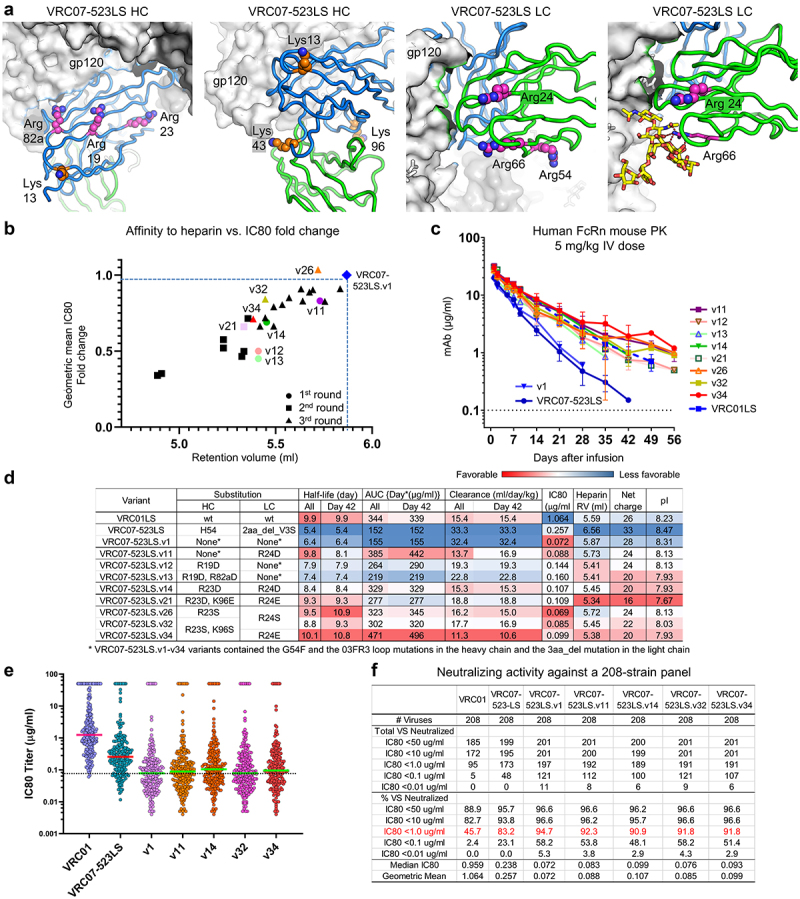
(a) Arg and Lys residues in the variable region of VRC07-523 selected for Asp, Glu, or Sersubstitution were demonstrated on the VRC07-523 Fab modeled using the VRC01.23-bound BG505 DS-SOSIP Env trimer (PDBID:6VI0). (b) VRC07-523LS charge variants were plotted according to the fold change of geometric mean IC80 values and the retention volumes on heparin column chromatography. (c) Pharmacokinetics of VRC07-523LS variants in human FcRn knock-in mice after administering a dose of 5 mg/kg intravenously. (d) PK properties, affinity to heparin, geometric mean IC80 fold changes against a 12-strain panel, and net charge of VRC07-523LS variants. The charge was calculated based on the sequence using Pepstats at https://www.ebi.ac.uk/Tools/seqstats/emboss_pepstats/ (e, f) Neutralizing activity of VRC07-523LS variants against a 208-strain panel.

To improve the half-life of VRC07-523LS.v1 by reducing the net positive charge in the variable domain, we first selected Arg19, Arg23, and Arg82a in the heavy chain and Arg24, Arg54, and Arg66 in the light chain based on ASA and information gained from a VRC07-bound gp120 structure (Figure 3a) and generated a panel of antibody variants incorporating Arg to Asp mutations (Kabat numbering used for amino acid positions). We then assessed their polyreactivity and neutralizing potency against a 12-strain panel (Supplementary Figure 1 and Supplementary Tables 1 and 2). All engineered variants showed reduced or no polyreactivity against HEp−2 cells and cardiolipin. Variants incorporating single substitutions in the heavy chain exhibited their potencies comparable to the parental, but particularly, variants incorporating Arg66 to Asp substitution (R66D) in the light chain showed substantially reduced potency (Supplementary Table 2). The basis for the reduced potency by R66D in the light chain was clear when we modeled Arg 66 in the cryo-EM structure of VRC01.23-bound BG505 DS-SOSIP^34^. It revealed Arg66 to be critical for VRC01 and VRC07 function as it interacts with the glycan at HIV−1 Env gp120 residue 276 (Figure 3a, right panel) via a salt bridge. This information allowed us to further refine our search for the best Args to mutate (Supplementary Table 2). After iterative optimization, we assessed the PK of VRC07-523LS.v11, v12, v13, and v14 in the human FcRn knock-in mouse model, as these four variants showed reduced affinity to heparin and the potencies comparable to the parental VRC07-523LS.v1 (Figure 3b). All four variants showed improved half-lives, area under the curve (AUC), and reduced clearance rates, of which VRC07-523LS.v11 and v14 showed half-lives improved to 9.8 days and 8.4 days from 6 days, AUC to 385 [day*(µg/mL)] and 329 from 155 and reduced clearance rates to 13.7 (mL/day/kg) and 15.3 from 32.5 at days 42 post-infusion, respectively (Figure 3c,d). [Two different groups of parameters were calculated: 1) using all animals at day 42 post-infusion including animals that have developed anti-drug antibody (ADA) responses, and 2) animals with no ADA response at day 42 post-infusion only (Figure 3d).] However, VRC07-523LS.v12 and VRC07-523LS.v13 did not show as much improvement in AUC and clearance rates as VRC07-523LS.v11 and VRC07-523LS.v14. VRC07523LS.v12 and VRC 07-523LS.v13 variants also had potencies reduced by more than the v11 and v14 variants (Figure 3d).

#### Second round- Screening of more variants incorporating Arg or Lys to Asp or Glu substitutions in the heavy and light chains

We next sought to improve VRC07-523LS.v11 and VRC07-523LS.v14 further by testing variants incorporating different combinations of Lys13 Lys43 or Lys96 to Glu substitution in the heavy chain (Figure 3a and Supplementary Table 3A). We tested variants incorporating double mutations or triple mutations in the heavy chain paired with Arg24 to Asp or Glu substitution in the light chain for their affinity to heparin and neutralizing potency. The four variants with R24E in the light chain (VRC07-523LS.v19 through VRC07-523LS.v22) showed the same trend toward affinity to heparin and potency as the variants with R24D but with slightly more reduced affinity to heparin and potency, although the results were not statistically significant (Supplementary Table 3A). Interestingly, variants with R24E mutation in the light chain exhibited better expression yields in transient transfection than their counterparts with R24D in the light chain (Supplementary Table 4). Therefore, we selected VRC07-523LS.v21 for PK assessment over VRC07-523LS.v17 as the VRC07-523LS.v21 variant showed similar properties on affinity to heparin and IC80 fold changes but with better expression yields. In the human FcRn mouse PK study, the half-life of 9.3 days for VRC07-523LS.v21 was slightly increased from 8.4 days relative to VRC07-523LS.v14 (Figure 3c,d) but other PK parameters such as AUC and clearance rates were not better than that of VRC07-523LS.v14. Thus the results led us to optimize further variants incorporating Arg23 and Lys96 in the heavy chain and Arg24 in the light chain (Supplementary Table 3A).

#### Third round- Screening of variants incorporating Arg or Lys to Glu Gln or Ser substitutions to optimize potency and affinity to heparin

Next we tested a panel of antibody variants containing Arg23_HC_ Lys96_HC_ and Arg24_LC_ replaced with uncharged but polar amino acids Gln or Ser and compared them to the Glu mutation. We found the order of Ser >Gln>Glu substitution for neutralizing potency from high to low and the order of Glu>Gln>Ser substitution for affinity to the heparin from low to high by a slight margin in general (Supplementary Table S3A). VRC07-523LS.v26 maintained potency with a modest decrease in affinity to heparin (Supplementary Table 3A). VRC07-523LS.v32 and VRC07-523LS.v34 variants exhibited the most reduced affinity to heparin with potencies comparable to the parental (Figure 3b and Supplementary Table 3A). The clearance rates of VRC07-523LS.v26, VRC07-523LS.v32, and VRC07-523LS.v34 in the human FcRn mice were decreased at least two-fold compared to their parental VRC07-523LS.v1 while their potencies retained close to the parental (Figure 3d). Overall, the PK parameters of the engineered variants were inversely correlated with their affinities with heparin (Supplementary Figure 1c).

In summary, our iterative optimization of electrostatic potentials in the variable domain identified VRC07-523LS.v11, VRC07-523LS.v14, VRC07-523LS.v26, VRC07-523LS.v32, and VRC07-523LS.v34 that exhibited improved PK parameters (Figure 3c,d) with their potencies comparable to the parental VRC07-523LS.v1. VRC07-523LS.v11, v14, v32, and v34 neutralized ~92% of the 208-virus panel with a geometric mean IC80 < 1 µg/mL (Figure 3e,f). VRC07-523LS.v34 showed the lowest affinity to heparin with neutralizing potency comparable to VRC07-523LS.v1 and exhibited the most improved PK parameters: half-life of 10.1 days and a clearance rate of 11.3 mL/day/kg from 6.4 and 32.4, respectively (Figure 3c,d). Moreover, VRC07-523LS.v34 showed favorable biophysical properties for manufacturing (Supplementary Table 6) and an estimated half-life in humans of ~59 days, a 55% increase from the half-life of 38 days for VRC07-523LS^35^ (Supplementary Figure 2).

### *A panel of N6LS antibody variants incorporating Arg or Lys to Asp Gln Glu or Ser substitutions in the variable domains identified variants with improved* in vivo *half-life*

#### First round - Screening of variants incorporating Arg to Asp substitutions based on ASA and an N6-bound gp120 structure

**Figure 4.**
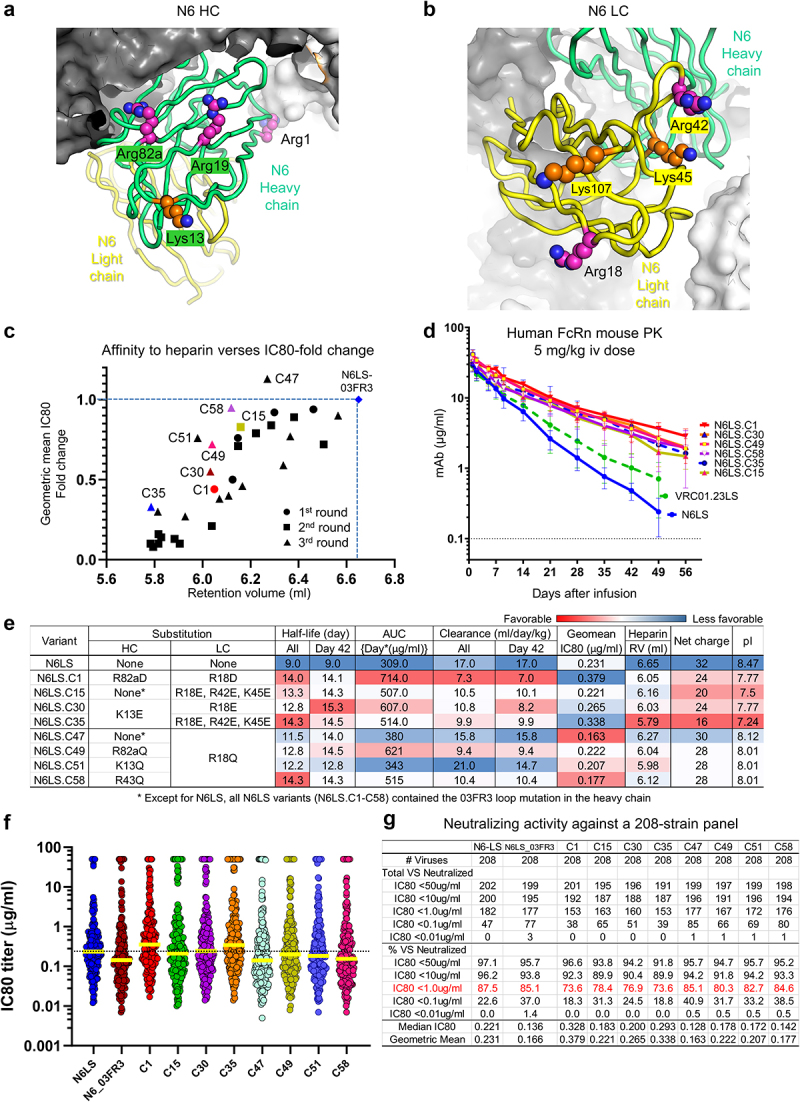
N6LS variants with select Arg or Lys to Asp Gln or Glu substitutions showed improved pharmacokinetics. (a,b) Arg and Lys residues in the variable region of N6LS selected for Asp Gln or Glu substitution were shown in surface representation to the N6LS Fab modeled using VRC01.23-bound BG505 DS-SOSIP Env trimer (PDB ID:6VI0). (c) N6LS charge variants were plotted according to the geometric mean IC80 fold change and the retention volumes on heparin column chromatography. (d) Pharmacokinetics of N6LS variants in human FcRn knock-in mice after injecting a dose of 5 mg/kg intravenous. (e) PK properties affinity to heparin IC80 fold changes against a 12-strain panel and net charge of N6LS variants. (f,g) Neutralizing potency against a 208-strain panel.

Encouraged by the improvement of VRC07-523LS variants, we sought to improve the PK of antibody N6, another CD4-binding site HIV−1 antibody. First, we selected Arg1, Arg19, and Arg82a in the heavy chain and Arg18 in the light chain for Asp substitution as these residues were highly exposed and did not interact with epitopes (Figure 4a,b). N6LS.C1, which incorporated Arg82a to Asp mutation in the heavy chain and Arg18 to Asp mutation in the light chain, showed the greatest reduction in affinity to heparin (Figure 4c and Supplementary Table 3B), and its half-life and AUC of the concentration versus time profile increased to 14 from 9 days and 714 to 309 [days*(µg/mL)], respectively. Its clearance rate decreased to 7 from ~17 mL/day/kg (Figure 4d,e).

#### Second round - Screening of more variants incorporating Arg or Lys to Glu mutations in the heavy and light chains

The PK parameters of N6LS.C1 were substantially improved. However, its potency was reduced more than 2-fold. Therefore, we tested more variants incorporating diverse Arg or Lys residues substituted in the heavy and light chain to find variants with improved PK and retained potency (Supplementary Table 3b). Notably, the affinities of engineered N6LS variants to heparin and their neutralizing potencies were substantially affected by Arg or Lys residues substituted in the heavy chain while Arg or Lys residues mutated in the light chain were tolerated. N6LS.C15 and N6LS.C16 incorporating three residues substituted in the light chain retained potency with reduced affinity to heparin. Notably, N6LS.C15 showed improved PK parameters: half-life, AUC, and clearance rate of 13.2 days, 507 [days*(µg/mL)], and 10.5 mL/day/kg, respectively (Figure 4e), with its potency retained more closely to the parental N6LS.

#### Third round - Screening and optimization of variants incorporating Arg of Lys to Glu substitutions in the heavy chain

Next, as N6LS.C15 contained Arg or Lys residues mutated in the light chain only, we tested whether the addition of K13, R19, R43, or R82a to Glu substitution in the heavy chain could further optimize the PK parameters and potency (Supplementary Table 3B). The addition of single substations to the heavy chain further reduced affinity to heparin but with reduced potency. N6LS.C30 incorporating K13E in the heavy chain reduced affinity to heparin most among the single substitutions in the heavy chain paired with R18E substitution in the light chain with its potency reduced less than two-fold. N6LS.C35 showed the most reduced affinity to heparin among all N6LS variants tested but its potency was reduced more than two-fold (Figure 4c). The two variants exhibited improved PK parameters: improved half-lives of ~14–15 days from 9 days and reduced clearance rates of ~8–10 mL/day/kg from 17 mL/day/kg (Figure 4d,e and Supplementary Figure 2B). However, addition of single substitutions in the heavy chain reduced potency.

#### Comparison of N6LS variants incorporating Arg or Lys to Asp Gln Glu or Ser substitution

As we narrowed down the residues to mutate in the heavy and light chains, we tested variants containing Arg or Lys to Asp, Gln, Glu, or Ser substitution and compared their effects on potency and affinity to heparin (Supplementary Table 3B). Overall, Ser or Gln substitutions had less of an impact on potency and affinity to heparin than Asp or Glu substitutions as shown in VRC07-523LS variants. Testing of these variants enabled us to identify N6LS.C47, C49, C51, and C58, which showed minimally reduced potencies and improved PK parameters compared to N6LS. Notably, C49 and C58 showed clearance rates that decreased to ~9 and ~10 from ~17 mL/day/kg with potency retained (Figure 4d,e).

In summary, N6LS.C1, C15, C30, and C35 variants showed improved PK parameters (Figure 4d,e) and neutralized 73%, 78%, 77%, and 73% of 208 strains at a geometric mean IC80 < 1 µg/mL, respectively, (Figure 4f-g). The potency-improved variants N6LS.C47, C49, C51, and C58 neutralized 85.1%, 80.3%, 82.7%, and 84.6% at a geometric mean IC80 less than 1 µg/mL, respectively, close to 87.5% for the parental N6LS (Figure 4e-g). Furthermore, N6LS.C49 and N6LS.C58 showed improved half-lives of ~14 days from 9 days for N6LS in human FcRn mice (Figure 4d). Our linear regression model estimated the half-life of the N6LS variants in humans to range from 71 to 85 days (Supplementary Figure 4) a 61% to 93% increase from 44 days for N6LS. Thus, these N6LS variants demonstrated that Arg or Lys to Gln or Ser substitution is preferable to improve PK than Asp or Glu substitution when the latter accompanies a significant loss in potency.

### Antibody binding affinity to heparin correlated with its affinity to FcRn at pH 7.4 when the Fab region is free to interact with the carboxymethyl-dextran matrix on the biosensors

**Figure 5.**
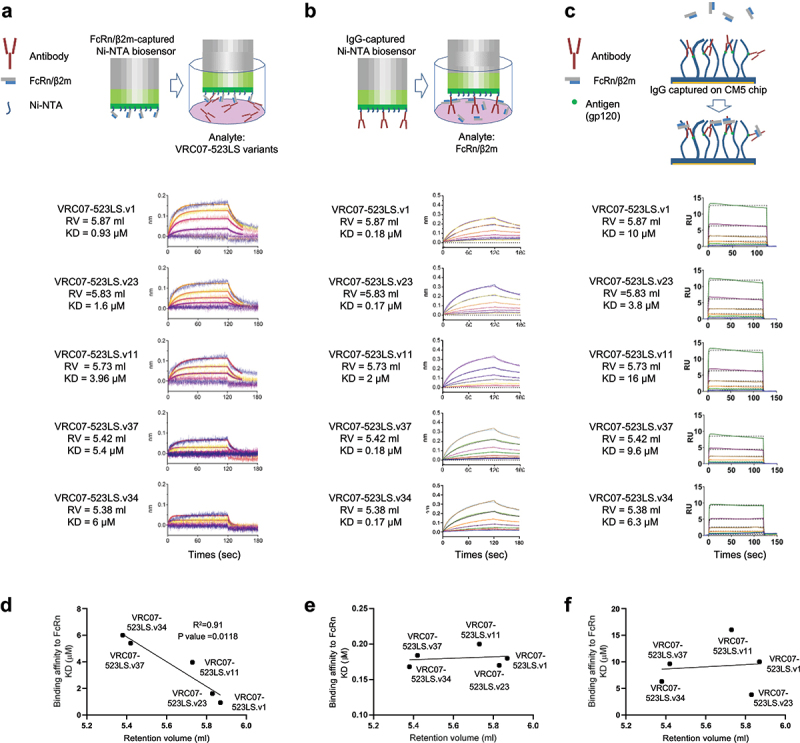
Binding affinities of VRC07-523LS variants to heparin correlated with their binding affinities to FcRn at pH 7.4 when the Fab regions were free to interact with the matrix of biosensors. (a) Ni-NTA biosensors coated with FcRn/β2m were dipped into wells containing VRC07-523LS variants. (b) Ni-NTA biosensors coated with VRC07- 523LS variants were dipped into wells containing FcRn/β2m. (c) FcRn/β2m was passed over VRC07-523LS variants captured by gp120 core immobilized on a CM5 chip. (d) the plot of the KDs measured in (A) vs. the retention volume on harpin chromatography. (e) the plot of the KDs measured in (b) versus the retention volume on harpin chromatography. (f) the plot of the KDs measured in (c) versus the retention volume on harpin chromatography.

To determine if the reduced net positive charge in the variable domain affects antibody-binding affinity to FcRn at pH 7.4, we measured the binding affinity of variants to FcRn using surface plasmon resonance (SPR) and bio-layer interferometry (BLI) in three different formats. In the first format, a BLI setup in which Ni-NTA biosensors coated with his-tagged FcRn were dipped into the wells containing VRC07-523LS variants, as a mimic of antibody Fc and FcRn interaction in physiological conditions. In this setup, the Fab region was free to interact with FcRn or the matrix on the biosensor, as a surrogate of the membrane components (Figure 5a). The second format used a BLI setup in which Ni-NTA sensors coated with his-tagged VRC07-523LS variants were immersed into the wells containing FcRn to enable the Fab regions to interact with FcRn without constraining the flexibility of IgGs (Figure 5b). In the third format, an SPR setup was used in which a range of concentrations of FcRn was passed over the VRC07-523LS variants captured by gp120 antigen immobilized on a CM5 chip to measure Fc-FcRn interaction only while avoiding any potential Fab-FcRn or Fab-matrix interactions (Figure 5c). The affinity of the variants to FcRn measured with the first format was observed to be in the rank order of VRC07-523LS.v1>VRC07-523LS.v23>VRC07-23LS.v34>VRC07-523LS.v37 from the highest to lowest which mirrored the rank order of variant-binding affinity to heparin (Figure 5d). However, binding affinities of VRC07-523LS variants to FcRn measured with the second and third formats did not show any significant differences among tested variants (Figures 5e,f). This demonstrates that reduced net positive charge contributes to half-life extension most likely by decreasing in absorption through pinocytosis and by facilitating the release of antibodies into the bloodat physiological pH, as a result of reduced off-targeting binding between antibody and endothelial cells.

### As Arg and Lys are highly prevalent at select positions in the variable domain and their positions are relatively conserved, substitution of these residues with net positive charge-reducing amino acids has the potential to increase the PK of human antibodies

**Figure 6.**
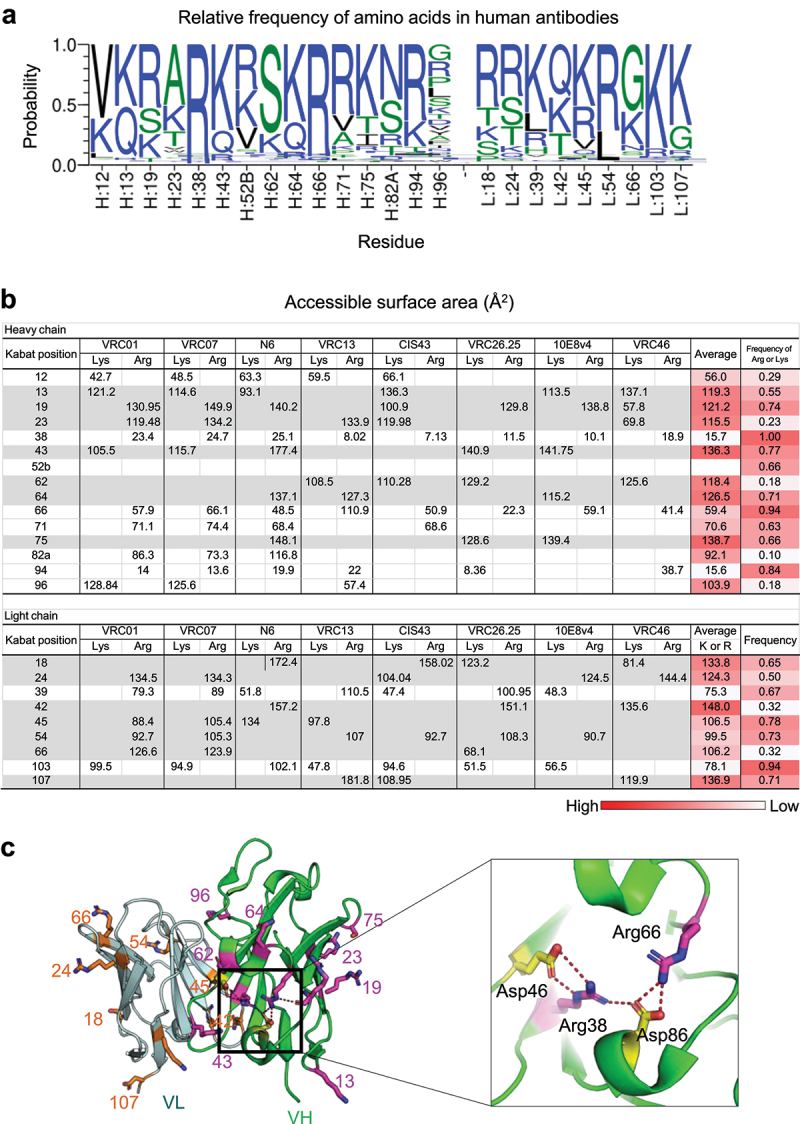
Arg or Lys is highly prevalent among amino acids at select positions in the variable region of human antibodies. (a) the relative frequency of amino acid in the variable region of human antibodies where Arg or Lys is the most or highly prevalent (abYsis server at http://www.abysis.org/abysis/searches/distributions/distributions.cgi. (b) Accessible surface areas were calculated from the structures of eight HIV−1 antibodies. Arg or Lys residues with high ASA and high relative frequency were highlighted in gray background. (c) Arg or Lys residues highly prevalent in human antibodies were shown in stick representation. Close-up view of four Arg or Lys that interact with neighboring Asp via salt bridges.

To assess the general applicability of reducing net positive charge in improving PK, we used the abYsis database (http://www.abysis.org/abysis/searches/distributions/distributions.cgi)^36^ to obtain the relative frequency of amino acids in the variable domain of human antibodies and found Arg and Lys to be most prevalent among other amino acids at positions: 13, 19, 23, 38, 43, 52B, 62, 64, 71, 75, 82A, 94, and 96 on heavy chain and 18, 24, 39, 42, 45, 54, 66, 103, 107, and 108 on light chain with the relative frequency of either Arg or Lys from ~18% to ~99% (Figure 6a and Supplementary Figure 2). The relative frequency of Arg or Lys among HIV−1 antibodies trended with that of human antibodies (Supplementary STable 5). Next, to evaluate if these residues were suitable for substitution with negatively charged or uncharged but polar residues without compromising structural integrity, we calculated the ASA of these residues by using the structures of eight different HIV−1 antibodies and found heavy chain Arg38 and Arg66, which are conserved 99% and 94% to be substantially buried with ASA of ~16 Å^2^ and ~59 Å^2^, respectively (Figure 6b). Furthermore, Arg38 interacted with Asp46 and Asp86 and Arg66 interacted with Asp86 via salt bridges (PDB ID: 4OLW) (Figure 6c). Interestingly, Asp 46 and Asp86 were also highly conserved (relative frequencies of 96% and 99%, respectively) in human antibodies, indicating a four-residue interaction to be critical for the structural integrity of the heavy chain and therefore, substitutions that alter these interactions would result in loss of function. We also found either Arg or Lys to be prevalent and exposed at positions 13, 19, 23, 43, 62, 64, and 75 on heavy chain and 18, 24, 42, 45, 54, 66, and 107 on light chain in human antibodies (Figure 6a,b). Notably, VRC07-523LS and N6LS variants incorporating Arg or Lys to Asp, Gln, Glu, or Ser mutations at positions Arg18, Arg24, Arg42, and Lys45 on light chain and Lys13, Arg19, Arg23, and Lys96 on heavy chain showed improved PK. Together, the high prevalence of Arg/Lys at select positions in the human antibodies and the identification of VRC07-523LS and N6LS variants with improved PK (Supplementary Table 7) suggest that substitution of these Arg or Lys to Asp, Gln, Glu, or Ser may serve to improve the PK of human antibodies in general.

### Estimated half-lives of VRC07-523LS and N6LS variants in humans

Significant correlations have been reported between terminal half-life or clearance rates observed in hFcRn mice and humans.^37,38^ Plasma concentration was modeled in humans using two-compartment analysis $C\left(t\right)=ae^{-\alpha t}+be^{-\beta t}$ where the first half of the function describes the initial phase and the second half describes the terminal phase. The b and β parameters were scaled allometrically using hFcRn mouse and human clinical data (Supplementary Figure 4A-C). This modeling approach estimated half-lives in humans ranging from 36 to 60 days for VRC07-523LS and 71 to 85 days for N6LS (Supplementary Figure 4D).   When we extrapolated the remaining concentrations of VRC07-523LS.v26 and VRC07-523LS.v34 after 6 and 12 months, they remained ~7-fold and ~50-fold higher relative to VRC07-523LS, respectively. Moreover, VRC07-523LS.v34 neutralized ~92% of 208 viruses at a geometric mean IC80 less than 1 µg/mL while VRC07-523LS neutralized ~83%. Similarly after 6 and 12 months, N6LS.C30, N6LS.C35, and N6LS.C49 had estimated increases of ~3-fold and ~6- to 7-fold relative to N6LS, respectively (Supplementary Figure 4D). Thus, these half-life estimations confirm that the PK and potency improved variants are appealing to reduce frequent dosing for viral suppression and antibody-mediated protection.

## Discussion

Here, we determined the basis of the reduced polyreactivity and increased *in vivo* half-lives of select VRC01-class antibodies to be reduced net positive charge due to four Asp residues incorporated by a 03FR3 loop insertion. We applied this finding to engineer highly potent VRC07-523LS and N6LS antibody variants with improved PK while retaining high functional potency. We assessed reduced net positive charge by using heparin affinity chromatography, polyreactivity, and neutralizing potency to screen a panel of antibody variants for improved PK.

We derived five key findings from this study, which may generally be applicable to improve the PK of other therapeutic antibodies. First, VRC07-523LS and N6LS variants incorporating Arg or Lys mutated to Asp, Gln, Glu, or Ser showed reduced affinity to heparin in the rank order of Glu>Asp>Gln>Ser mutation in general with Glu being most reduced (Supplementary Table 3A). Second, the reduced affinity to heparin, however, resulted in reduced neutralizing potency, which also mirrored the rank order of reduced affinity to heparin: Glu>Asp>Gln>Ser (Supplementary Figure 3D). Third, the same Arg or Lys to Asp or Glu substitutions exhibited different degrees of affinity for heparin and potency losses depending on where they were located. For example, K43E incorporated in VRC07-523LS.v16 and VRC07-523LS.v20 heavy chain exhibited a more reduced affinity to heparin and reduced potency than K13E and K96E (Supplementary Table 3A). Fourth, engineered VRC07-523LS variants with substitutions in the light chain exhibited a more drastic change in affinity to heparin and potency, while the affinity of N6LS variants to heparin and their neutralizing potencies were affected more substantially by substitutions in the heavy chain, indicating that hotspots for improving PK are antibody dependent. Fifth, the VRC07-523LS and N6LS variants’ affinities to heparin inversely correlated with improvement in PK (Supplementary Figure 3C) but with no statistical significance in part due to a relatively small sample size. Nonetheless, VRC07-523LS and N6LS variants with these substitutions significantly improved PK properties compared to their parentals (Supplementary Figure 3A and B). However, all charge substitutions tested resulted in a different degree of potency loss. Therefore, to successfully implement this approach, it may be necessary to screen Arg or Lys residues that affect potency minimally when substituted with either Asp or Glu.

Overall, Arg or Lys to Glu or Asp substitutions resulted in substantial reduction in affinity to heparin and potency followed by Gln and Ser substitutions (Supplementary Figure 3D). Depending on the magnitude of loss in potency, Asp or Glu can be replaced with uncharged but polar amino acids such as Asn, Gln, Thr, or Ser to minimize potency losses. We found that variants with Gln or Ser substitutions preserved potency more closely than variants with Asp or Glu substitutions (Supplementary Figure 3D). For example, triple Ser substitutions enabled VRC07-523LS.v32 to reduce its affinity to heparin by the same magnitude as that of VRC07-523LS.v14 which contained two Asp substitutions but retained potency more closely to that of the parental than VRC07-523LS.v14 (Supplementary Figure 3D).

Interestingly, most variants with improved PK exhibited shifts of isoelectric point (pI) of less than a half unit. For example, a single mutation R24D in the light chain of VRC07-523LS.v11, with a shift of only 0.18 units, substantially increased PK while preserving potency and breadth (Figure 3d). Arg18 to Asp mutation, R18D, in the N6 light chain in N6LS.C1 also improved PK of N6LS.C1 when combined with heavy chain R82aD mutation with the shifts in pI less than a half unit (Figure 4e). In line with our data, Schoch et al.^19^ also observed that engineered antibody variants with positively charged residues mutated in the variable domain (Fv) show altered affinity to FcRn with only a narrow range of differences in pI values, less than 1 unit. They proposed that the altered PK is caused by reduced Fv-FcRn binding at physiological pH mediated by specific residues in the Fv rather than by reduced endocytosis resulting from the reduced off-target bindings between the Fv and endothelial cells. Boswell et al.^39^ had previously proposed that measurable PK change due to altered off-target binding between IgG and endothelial cells can occur with shifts in pI values of at least one unit. Structural evidence of direct Fab-FcRn contacts in a physiologically relevant environment, however, is lacking although previous studies using electron tomography^40,41^ molecular dynamics simulations^19^ or hydrogen/deuterium exchange mass spectrophotometry^42,43^ suggested the possibility of the direct interactions between Fab and FcRn. Piche-Nicolas et al.^16^ used SPR to measure direct Fab-FcRn interaction but did not observe any measurable binding in the absence of primary binding between the Fc and FcRn. Furthermore, the presence of carboxymethyl dextran matrix influences the KDs of variants to FcRn^16^ suggesting that the positively charged variable region interact with the negatively charged matrix.

We also failed to observe direct electrostatic interactions between Fv and FcRn (Figure 5b,f). Rather, our data suggest that the reduced net positive charge in the FR of the variable domain may contribute to the improved PK by reducing endocytosis as well as the Fv-FcRn interaction at a physiological pH resulting from altered electrostatic interaction between Fv and the endothelial membranes around FcRn. Neuber et al.^44^ also reported that the variations in the FRs or the complementarity-determining regions (CDRs) of the Fab domain did not significantly change FcRn binding. Therefore, further studies are warranted to determine the direct interaction between variable domain and FcRn.

While previous studies have demonstrated that the CDRs in the variable domain but not the FR play a role in modulating Fab and FcRn interaction,^16^ we observed a patch of negatively charged residues introduced in the FR, as a result of engrafting the 03FR3 loop to VRC01-class antibodies, to reduce polyreactivity and to improve PK. We also engineered VRC07-523LS and N6LS variants incorporating select Arg and Lys mutated to Asp, Gln, Glu, or Ser in the FR to show improved PK. Furthermore, the engineered variants containing the Arg or Lys at positions 18 and 24 substituted to Asp or Glu substitutions in the light chain improved the PK of VRC07-523LS and N6LS substantially, and the relative frequency of the two residues were observed to be 57% and 51%, respectively, among ~35000 antibodies (Figure 6a and Supplementary Figure 2), suggesting that the same mutations could potentially improve the PK of other antibodies. Our findings taken together with the observation that Arg or Lys is highly prevalent at select positions in the FR of the variable domain (Figure 6) and the effects of charge on the plasma PK of antibodies reported in the literature^17–19,45^ demonstrate that reducing net positive charge in the FR of the variable domain could serve as a promising way to improve the PK of therapeutic antibodies with preserved potency.

## Materials and Methods

### Expression of antibody variants in Expi293 cells

Antibody variable heavy chain and light chain sequences were codon-optimized synthesized and cloned into a pVRC8400 (CMV/R expression vector)-based IgG1 vector as previously described.^46^ The variants were expressed by transient transfection in Expi293 cells (Thermo Fisher Scientific) using Turbo293 transfection reagent (SPEED BioSystems) according to the manufacturer’s recommendation. 50 µg plasmid encoding heavy-chain and 50-µg plasmid encoding light-chain variant genes were mixed with the transfection reagents added to 100 mL of cells at 2.5 × 10^6^/mL and incubated in a shaker incubator at 120 rpm 37°C 9% CO_2_. At 5 days post-transfection cell culture supernatant was harvested and purified with a Protein A (GE Healthcare) column. The antibody was eluted using IgG Elution Buffer (Thermo Fisher) and was brought to neutral pH with 1 M Tris-HCl pH 8.0. Eluted antibodies were dialyzed against phosphate-buffered saline (PBS) overnight and were confirmed by SDS-PAGE before use.

### HEp−2 cell staining and cardiolipin ELISA assay

Polyreactivity was determined by ANA HEp−2 Staining Analysis (ZEUS Scientific Cat. No: FA2400) and anticardiolipin ELISA (Inova Diagnostics Cat. No.: 708625). For the HEp−2 assay all antibodies were tested at 25 and 50 μg/mL per manufacturer’s protocol and imaged on a Nikon Ts2R microscope for 500 ms. Scores from 0 to 3 were defined with four control antibodies VRC01-LS 4E10 VRC07-523LS and VRC07-G54W. Test antibodies were scored by visual estimation of staining intensity compared to the control antibodies. Scores equal to or greater than 1 at 25 μg/mL were classified as polyreactive and between 0 and 1 as mildly polyreactive. In the cardiolipin, ELISA antibodies were tested at a starting concentration of 100 μg/mL followed by 3-fold dilutions. IgG phospholipid (GPL) units were calculated from the standard curve. GPL score < 20 was considered as not reactive 20–80 as low positive and > 80 as high positive.

### Heparin affinity chromatography

Each antibody sample was diluted in 1500 µl of mobile phase A (MPA) 10 mM sodium phosphate pH 7.2 ± 0.2 to a final concentration of approximately 20 µg/mL. It was then injected onto the HiTrap 1 mL Heparin HP column (Cytiva Life Sciences Marlborough MA) on a BioRad (Hercules CA) NGC Chromatography System Quest 10. The flow rate was set to 1.0 mL/min and the mobile phase B (MPB) was 10 mM sodium phosphate 1 M NaCl pH 7.2 ± 0.2. The column was equilibrated in 100% MPA before each injection; the gradient was (1): 0-2 min 100% MPA; (2): 2–12 min 100% MPA to 100% MPB; (3) 12–14 min 100% MPB. UV absorbance was detected at 280 nm using Chromlab.

### Neutralization assay

Single-round-of-replication Env pseudoviruses were prepared titers were determined and the pseudoviruses were used to infect TZM-bl target cells as described previously.^47^ Neutralization of monoclonal antibodies (mAbs) was determined using a multiclade panel of 12 HIV−1 Env-pseudoviruses including clade A (2) clade AG (1) clade B (4) clade C (4) and clade D (1) and using a 208-isolate panel.^48^ Each mAb was assayed at 5-fold dilutions starting at 50 μg/mL. The neutralization titers were calculated as a reduction in luminescence units compared with control wells and reported as 50% or 80% inhibitory concentration (IC_50_ or IC_80_) in micrograms per milliliter.

### Human FcRn knock-in mouse PK

Human FcRn transgenic mice (C57BL/6 B6.mFcRn−/− hFcRn Tg32 line from The Jackson laboratory) were used to assess the PK of VRC07-523LS and N6LS antibody variants. Each animal was infused intravenously with 5 mg of mAb/kg of body weight. Whole blood samples were collected at day 1, 2, 5, 7, 9, 14, 21, 28, 35, 42, and 56. Serum was separated by centrifugation. Serum mAb levels were measured by ELISA as described previously.^27^ All mice were bred and maintained under pathogen-free conditions at an American Association for the Accreditation of Laboratory Animal Care (AAALAC)-accredited animal facility at the NIAID and housed in accordance with the procedures outlined in the Guide for the Care and Use of Laboratory Animals. All mice were between 6 and 13 weeks of age. The study protocol was evaluated and approved by the NIH Animal Care and Use Committee (ASP VRC−18-747). The PK parameters were calculated using the Phoenix WinNonlin software (Certara). Two different parameters for half-life and the clearance rates were calculated using: 1) data sets at day 42 post-infusion with no ADA responses to mAbs and 2) all data sets at day 42 post-infusion including ones from animals that developed ADA responses to mAbs before day 42. To calculate the AUC of the serum mAb concentration versus time profile data sets at day 42 post-infusion with no ADA responses were used.

### Amino acid frequency analysis

HIV antibody sequences were downloaded from GenBank on August 7, 2021 and kept the sequences identified as *Homo sapiens* for amino acids frequency analysis. The Kabat numbering was assigned to the sequences using standalone ANARCI.^49^ The sequences that could not be assigned Kabat numbering were removed from the analysis. An in-house python script was applied to calculate the frequency of amino acids from 6589 heavy chains and 7109 light chains of HIV antibodies. We used the AbYsis server to obtain the frequency of the amino acids of human antibodies (http://www.abysis.org/abysis/searches/distributions/distributions.cgi).^50^

### Fc-FcRn binding kinetics by surface plasmon resonance

The binding kinetics of VRC07-523LS variants to FcRn was measured using Biacore T200 (Cytiva). The FcRn/β2 m heterodimers in HBS-EP+ buffer (10 mM HEPES 7.4 150 mM NaCl 3 mM EDTA 0.005% v/v Surfactant P20) (Cytiva) in a two-fold dilution series from the highest concentration of 1 to 0 M were passed over the VRC07-523LS variants captured (~300 RU) by HIV−1 gp120 core_e_ clade A/E 93TH057^51^ immobilized onto a CM5 chip by amine coupling and monitored association for 120 s at a flow rate of 40 µL/min and dissociation for 60 s. In another format VRC07-523LS variants in HBS-EP+ buffer in a two-fold dilution series from the highest concentration of 500 nM were passed over the biotinylated FcRn/β2m heterodimer captured (~150 RU) on an SA chip at a flow rate of 40 µL/min. and monitored association for 120 s and dissociation for 120 s. The kinetics parameters were extracted by fitting the sensorgrams with the 1:1 Langmuir model using BIA evaluation software.

### Fc-FcRn binding kinetics by bio-layer interferometry

We used the Octet HTX system (ForteBio) to measure the Fc-FcRn binding kinetics. To assess the binding kinetics between the VRC07-523LS variants and FcRn/β2 m with FcRn/β2m as the ligand we presoaked Ni-NTA biosensors in PBS loaded them with His-tagged FcRn/β2m at a density of ~0.3 nm by immersing the biosensors in wells containing 125 nM of 6×His-tagged FcRn/β2m at the C-terminus of the β2m chain for 1 min. We then baselined the biosensors for 1 min at HBS-EP+ buffer pH 7.4 and measured the association by dipping the biosensors into wells containing VRC07-523LS variants at concentrations ranging from 0 to 1000 nM in HBS-EP+ buffer pH 7.4 for 2 min followed by dissociation at the same buffer for 1 min. As a reference, we repeated the entire process with blank Ni-NTA biosensors. The sensorgrams were double referenced and globally fitted with a 1:1 model to extract kinetic parameters. To assess the binding kinetics between VRC07-523LS variants and FcRn/β2m with the VRC07-523LS variants as ligands we captured the VRC07-523LS variants containing a 6×His-tag after a GGSG linker at the C-terminus of the heavy chain with Ni-NTA biosensors. We baselined the biosensors at HBS-EP+ buffer pH 7.4 for 1 min and measured the association for 2 min at wells of FcRn/β2m at concentrations ranging from 0 to 1000 nM in HBS-EP+ buffer pH 7.4 followed by dissociation at the same buffer for 1 min. The sensorgrams were referenced against the reference signal obtained in the absence of FcRn/β2 m before being globally fitted with a 1:1 model to extract kinetic parameters. The 6×His-tagged FcRn/β2m was purified by Ni-NTA affinity chromatography (cOmplete™ His-Tag Purification Resin Roche) and the 6×His-tag cleaved FcRn/β2m was generated by cleaving its 6×His-tag preceded by HRV 3C cleavage site at the C-terminus of the β2m chain with Pierce™ HRV 3C Protease Solution Kit (Thermo Fisher Scientific) according to the manufacturer’s protocol.

### Data analysis

ASA was calculated using PDBePISA.^52^ Distances of amino acids from respective epitopes were calculated using PyMOL(The PyMOL Molecular Graphics System Version 2.0 Schrödinger LLC). Net charge and the isoelectric point (pI) of variants were calculated using EMBOSS Pepstats^53^ at https://www.ebi.ac.uk/Tools/seqstats/emboss_pepstats/. Unpaired t-tests were computed using GraphPad Prism version 8 for Windows (GraphPad Software La Jolla California USA www.graphpad.com).

### Half-life estimation modeling

Linear regression was applied to model the mean terminal half-life in humans as a function of half-life in hFcRn TgM mice *µ*(*X*) = *β*_0_ + *β*_1_*X*. The intercept *β*_0_ and slope *β*_1_ were estimated using ordinary least squares. Statistical modeling was performed using the statsmodels and uncertainties open-source Python libraries. The ratios of the estimated mean half-life in humans relative to the half-life in hFcRn TgM mice were used to scale serum mAb levels for modeling of relative concentration estimates in humans over time. Two linear regression models were fit to the log-transformed scaled data to model the rapid absorption half-life t½(α) phase and slower elimination t½(β) half-lives phase.

## Supplementary Material

Supplemental Material

## Abbreviations

IgG: Immunoglobulin G
BLI: biolayer interferometry
KD: dissociation constant
Fab: antigen-binding fragment
Fc region: the fragment crystallizable region
PK: Pharmacokinetics
SPR: surface plasmon resonance
FcRn: neonatal Fc receptor
ELISA: enzyme-linked immunoassay
IV: intravenous
