# Supplementary material for: Improved pharmacokinetics of HIV-neutralizing VRC01-class antibodies achieved by reduction of net positive charge on variable domain
Source: MAbs. 2023 Jun 21;15(1):2223350. doi: 10.1080/19420862.2023.2223350 (PMC10288911; doi:10.1080/19420862.2023.2223350)
Supplement: Supplemental Material [file KMAB_A_2223350_SM4144.docx]

# Figure S1

No mAb VRC01-LS 4E10 VRC07-523LS VRC07-G54W

Controls


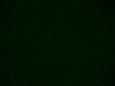

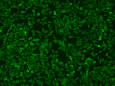


**0**

**1**


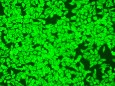


**2**


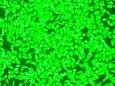


**3**


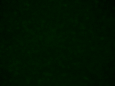


**0**

VRC07-523LS.v1

HC-R19D

HC-R23D

HC-R82aD

LC-R24D

VRC07-523LS.v1


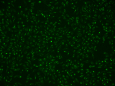

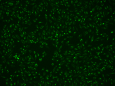

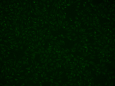


**0**

**0/1**

**0/1**


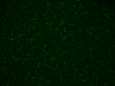


**0/1**


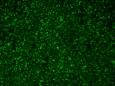


**1**

variants

LC-R54D

LC-R66D

LC-R24D+R66D LC-R24D+R54D

LC-R54+R66D

LC-R24+R54+R66D


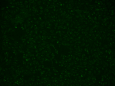


**0/1**


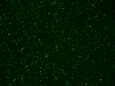


**0/1**


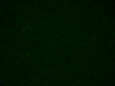

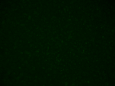


**0**

**0**


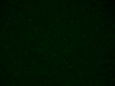


**0**


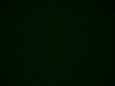


**0**

VRC07-523LS.v1 HC-R82aD/LC-R54D

VRC07-523LS.v1 HC-R82aD/LC-R24D

VRC07-523LS.v1 HC-R23D/LC-R54D

VRC07-523LS.v1 HC-R23D/LC-R24D

VRC07-523LS.v1 HC-R19D/LC-R24D

VRC07-523LS.v1 LC-R54D


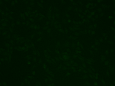

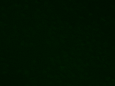

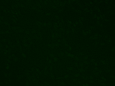

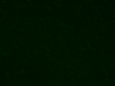

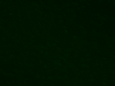

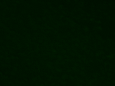


**0**

**0**

**0**

**0**

**0**

**0**

VRC01-LS 4E10 VRC07-523LS VRC07-G54W no mAb

VRC07-523LS.v1 HC-R19D+R23D


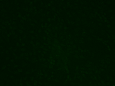

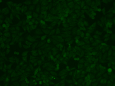

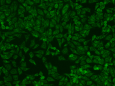

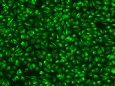

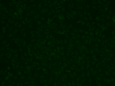

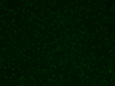


**0**

**1**

**2**

**3**

**0**

**0**

VRC07-523LS.v1 R23D,R82aD/R54D

VRC07-523LS.v1 R23D,R82aD/R24D

VRC07-523LS.v1

R23D, R82aD/3aa_del

VRC07-523LS.v1 R19D,R82aD/R24D

VRC07-523LS.v1 R19D+R82aD

VRC07-523LS.v1 R19D+R23D/R24D


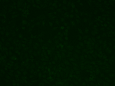

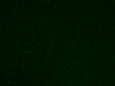

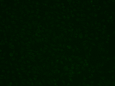

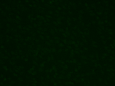

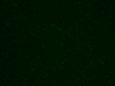

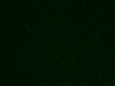


**0**

**0**

**0**

**0**

**0**

**0**

N6-LS

N6-03FR3-LS

N6-03FR3-LS.C1

N6-03FR3-LS.C2

N6-03FR3-LS.C3

N6-03FR3-LS.C5

###


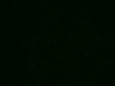

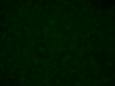


**0**

**0**


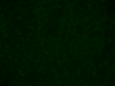

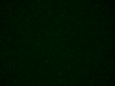


**0**

**0**


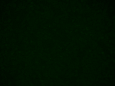


**0**


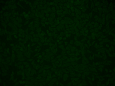


**0**

**Figure S1. Substitution of Arg with Asp reduced polyreactivity of VRC07-523LS and N6LS variants.** HEp-2 cell staining assay against VRC01 variants was performed at 25 μg/ml concentration along with control antibodies, VRC01-LS, 4E10, VRC07-523LS, and VRC07-G54W. Control antibodies were assigned a score between 0 and 3. Test antibodies scored greater than 1 at 25 µg/ml were considered polyreactive.

Supplementary Figure 1 Alt Text: HEp-2 cell staining assay against VRC01 variants was performed at 25 µg/ml concentration along with control antibodies, VRC01-LS, 4E10, VRC07- 523LS, and VRC07-G54W. Test antibodies scored greater than 1 at 25 µg/ml were considered polyreactive.

**A 80**


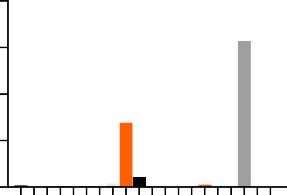


Kabat H12

**Relative Frequency (%)**

**60 60**


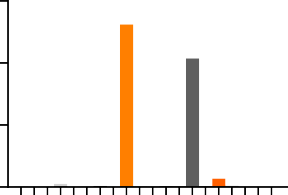


Kabat H13


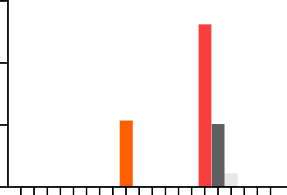


Kabat H19

**Relative Frequency (%)**

**Relative Frequency (%)**

# Figure S2

**60**


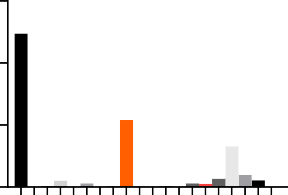


Kabat H23

**Relative Frequency (%)**

**60**

**40 40 40**

**40**

**20 20 20**

**20**

**0**

**A C DE FG H I K L M N P Q RS T V W Y**

**0**

**ACD E F G H I K LM N P Q S R T VW Y**

**0**

**A C DE FG H I K L M N P Q RS T VW Y**

**0**

**AC D E F G H I K L M N P Q R S T V W Y**

**100**

**Relative Frequency (%)**

**50**

**0**

Kabat H38


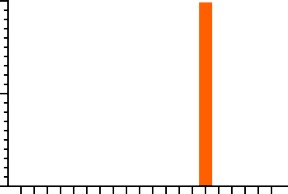


**A C DE FG H I K L M N P Q RS T VW Y**

**80** Kabat H43


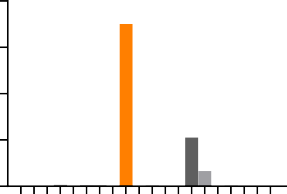
**60**

**Relative Frequency (%)**

**40**

**20**

**0**

**AC D E F G H I K L M N P Q R S T V W Y**

**40** Kabat H52B


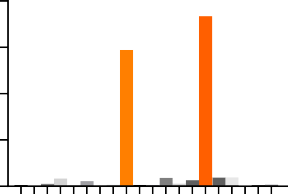
**30**

**Relative Frequency (%)**

**20**

**10**

**0**

**A C DE FG H I K L M N P Q RS T V W Y**

**80** Kabat H62


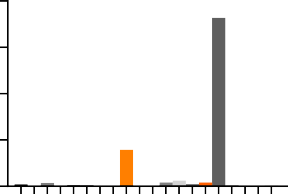
**60**

**Relative Frequency (%)**

**40**

**20**

**0**

**AC D E F G H I K L M N P Q R S T V W Y**


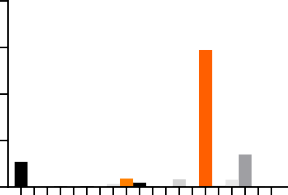

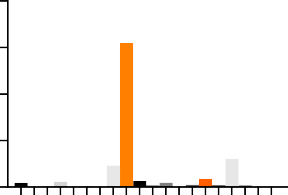
Kabat H64 Kabat H66 Kabat H71 Kabat H75

**Relative Frequency (%)**

**Relative Frequency (%)**

**Relative Frequency (%)**


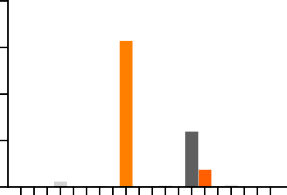
**80**

**Relative Frequency (%)**

**60**

**40**

**20**

**0**

**A C DE FG H I K L M N P Q RS T V W Y**

**100**

**80**

**60**

**40**

**20**

**0**


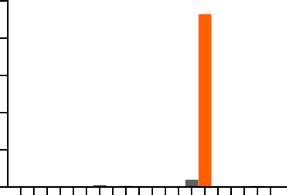


**A C DE F G H I K L M N P Q RS T V WY**

**80**

**60**

**40**

**20**

**0**

**A C DE FG H I K L M N P Q RS T V W Y**

**80**

**60**

**40**

**20**

**0**

**AC D E F G H I K L M N P Q R S T V W Y**

Kabat H82a


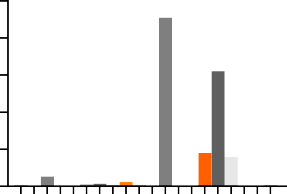
**50**

**Relative Frequency (%)**

**40**

**30**

**20**

**10**

**0**

**A C DE FG H I K L M N P Q RS T V W Y**

Kabat H94


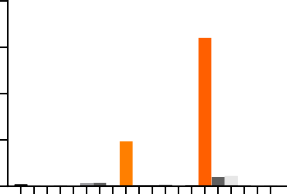
**80**

**Relative Frequency (%)**

**60**

**40**

**20**

**0**

**ACD E F G H I K L M N P Q R S T V WY**

Kabat H96


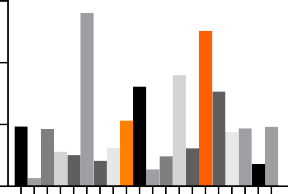
**15**

**Relative Frequency (%)**

**10**

**5**

**0**

**A C DE FG H I K L M N P Q RS T V W Y**

**B** Kabat L18 Kabat L24 Kabat L39 Kabat L42


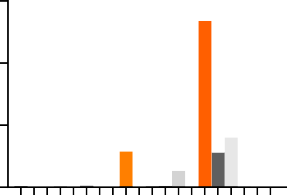
**60**

**Relative Frequency (%)**

**40**

**20**

**0**

**A C DE FG H I K L M N P Q RS T V W Y**

**50**


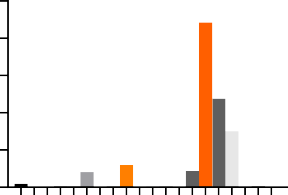
**40**

**Relative Frequency (%)**

**30**

**20**

**10**

**0**

**A C DE FG H I K L M N P Q RS T V W Y**

**80**


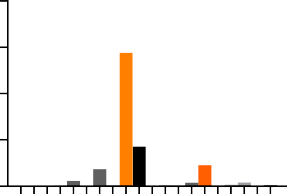
**60**

**Relative Frequency (%)**

**40**

**20**

**0**

**A C DE FG H I K L M N P Q RS T V W Y**

**50**


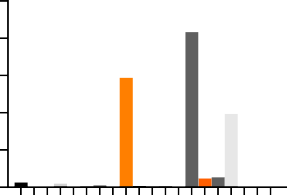
**40**

**Relative Frequency (%)**

**30**

**20**

**10**

**0**

**A C D E F GH I K L M N P QR S T VW Y**


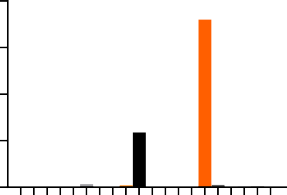
**80** Kabat L54

**Relative Frequency (%)**

**60**

**40**

**20**

**0**

**A C D E F GH I K L M N P QR S T VW Y**

**60** Kabat L66


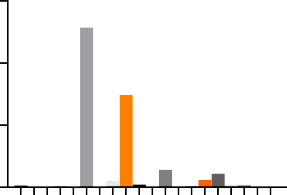
**40**

**Relative Frequency (%)**

**20**

**0**

**ACD E F G H I K L M N P Q R S T V WY**

**100**

**80**

**Relative Frequency (%)**

**60**

**40**

**20**

**0**

Kabat L103


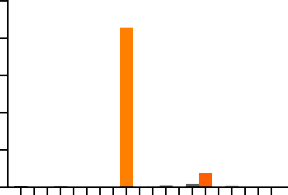


**A C DE F G H I K L M N P Q RS T V W Y**

**80** Kabat L107


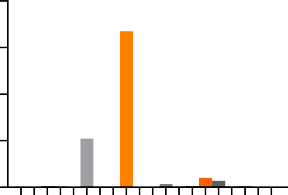
**60**

**Relative Frequency (%)**

**40**

**20**

**0**

**AC D E F G H I K L M N P Q R S T V W Y**

**Figure S2. Locations where Arg or Lys is most or highly prevalent are relatively conserved in the VH and VL regions of human antibodies.** (A) The VH region of human antibodies in Kabat numbering where Arg or Lys is the most or highly prevalent. (B) The VL region of human antibodies in Kabat numbering where Arg or Lys is the most or highly prevalent. The relative frequency of Arg and Lys obtained using the abYsis server at <http://www.abysis.org/abysis/searches/distributions/distributions.cgi>is shown in orange bars.

Supplementary Figure 2 Alt Text: Panel a. The VH region of human antibodies in Kabat numbering where Arg or Lys is the most or highly prevalent. Panel b. The relative frequency of Arg and Lys obtained using the abYsis server at <http://www.abysis.org/abysis/searches/distributions/distributions.cgi>is shown in orange bars.

# Figure S3

1. Half-life Area under the curve from serum mAb concentration vs time

profile

Clearance

P value = 0.09 P value = 0.05 P value = 0.0021


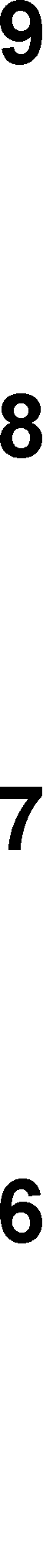

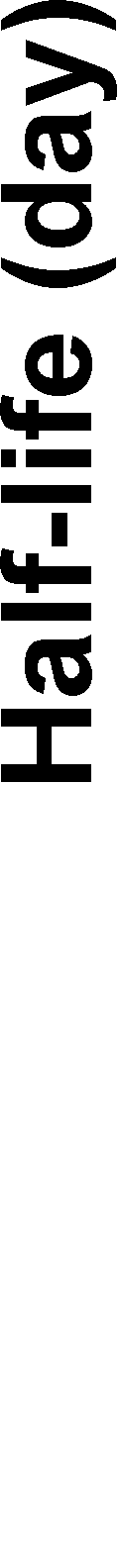

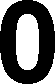

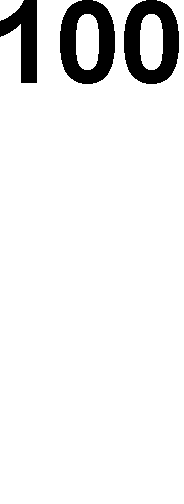

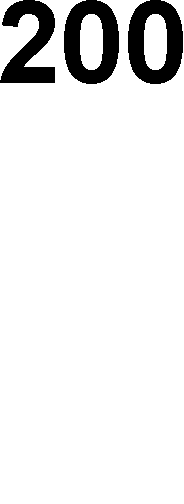

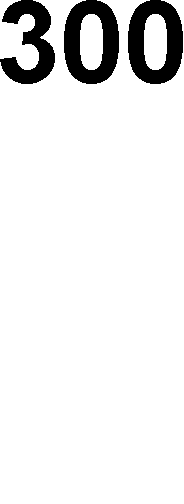

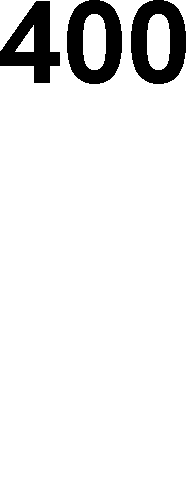

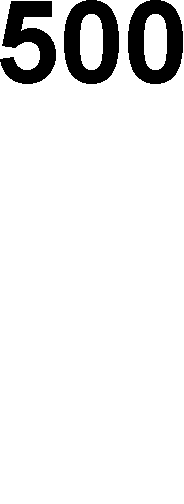

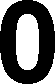

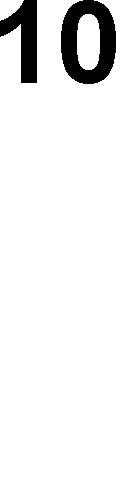

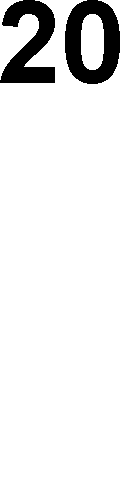

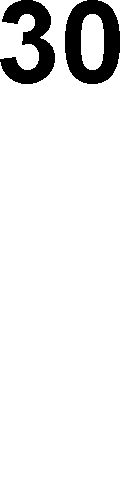

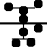

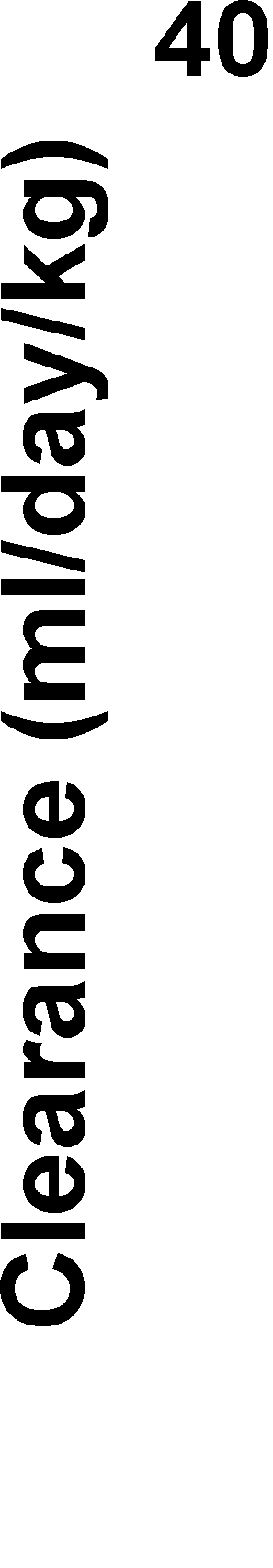


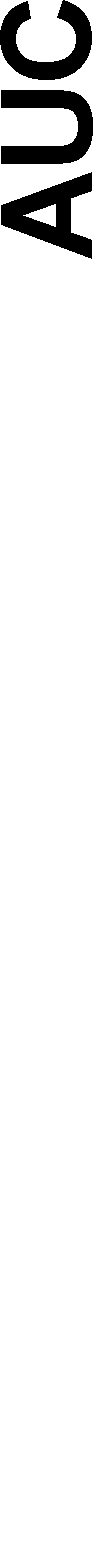


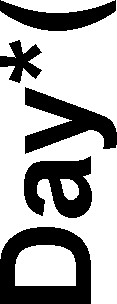

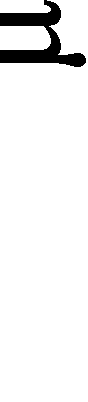

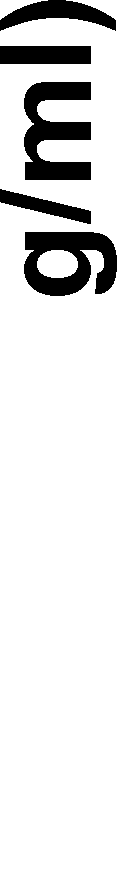


1. P value = 0.0086


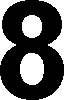

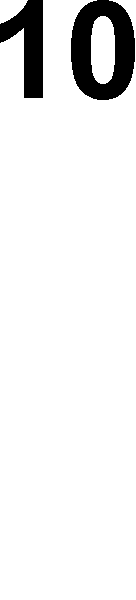

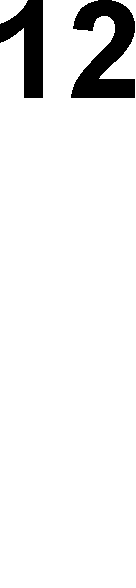

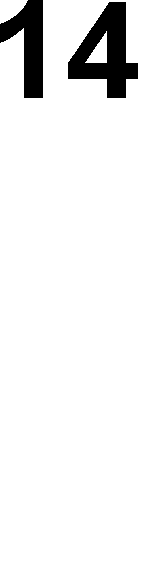

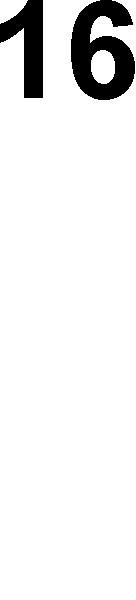

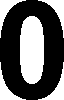

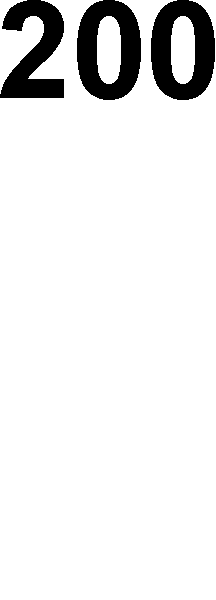

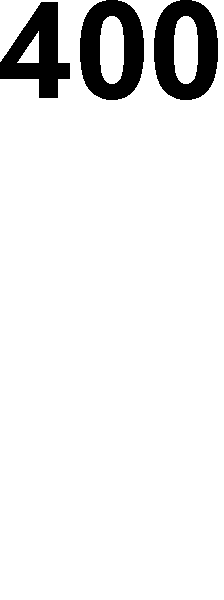

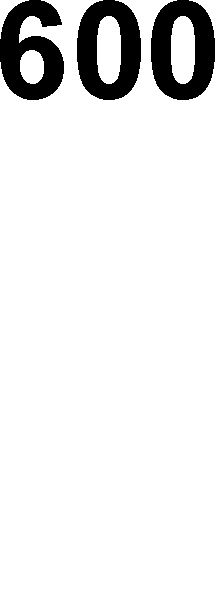

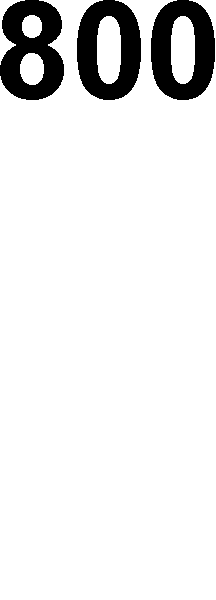

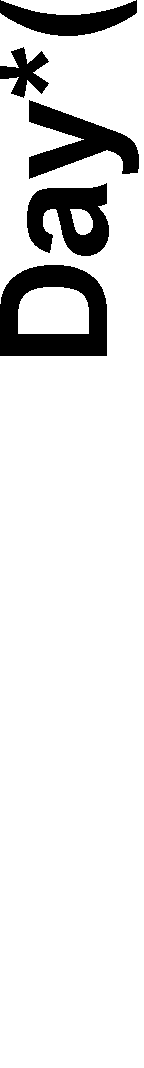

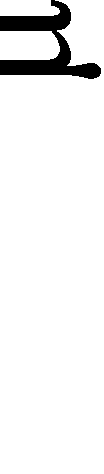

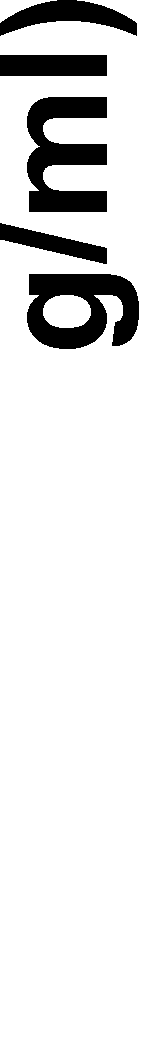


P value = 0.08

P value = 0.02


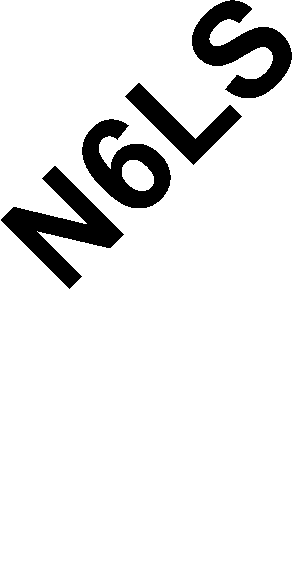

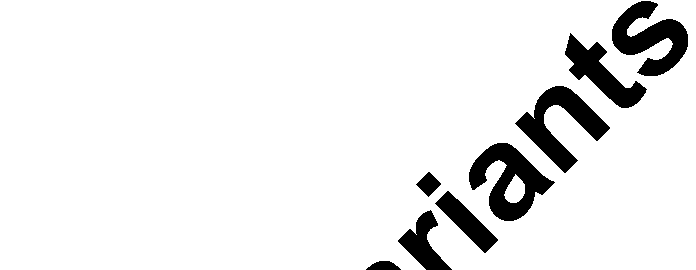

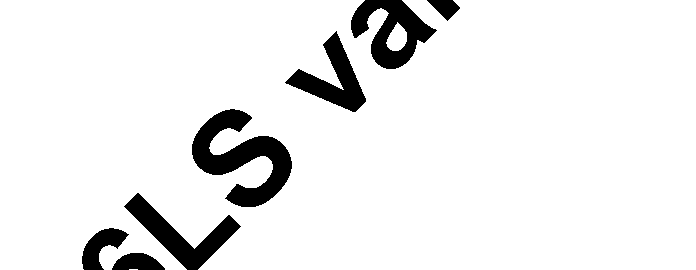

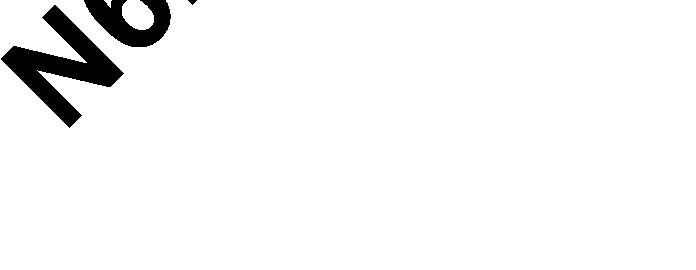

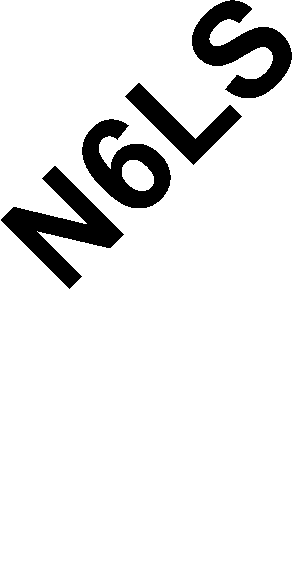

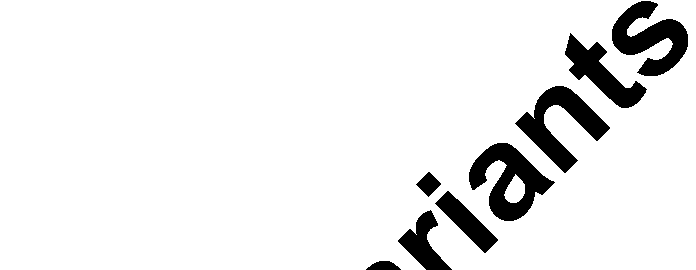


**Figure S3. VRC07-523LS and N6LS charge variants showed their PK parameters significantly improved compared to their parentals.** (A) Improved PK parameters -half-life, area under the curve of serum mAb concentration vs. time, and clearance - of VRC07-523LS charge variants. (B) Improved PK parameters -half-life, area under the curve of serum mAb concentration vs. time, and clearance- of N6LS charge variants. (A and B) *P* value was calculated using unpaired t-test with the 95% confidence interval

**C**

Retention volume vs Half-life

**D**

Retention volume vs IC80 fold change

Retention volume (ml)

Retention volume (ml)

*Charge was calculated based on sequence [https://www.ebi.ac.uk](http://www.ebi.ac.uk/Tools/seqstats/emboss_pepstats/)/[Tools/seqs](http://www.ebi.ac.uk/Tools/seqstats/emboss_pepstats/)tats[/emboss_peps](http://www.ebi.ac.uk/Tools/seqstats/emboss_pepstats/)t[ats/](http://www.ebi.ac.uk/Tools/seqstats/emboss_pepstats/)

Half-life (day)

Geometric mean IC80 Fold change

(C) Correlation between heparin column retention volume and half-life. (D) Affinity to heparin vs. geometric mean IC80 fold change of VRC07-523LS variants.

Supplementary Figure 3 Alt Text: Panel a. Improved PK parameters -half-life, area under the curve of serum mAb concentration vs. time, and clearance - of VRC07-523LS charge variants. Panel b. Improved PK parameters -half-life, area under the curve of serum mAb concentration vs. time, and clearance- of N6LS charge variants. P value was calculated using unpaired t-test with the 95% confidence interval. Panel c. Correlation between heparin column retention volume and half-life. Panel d. Affinity to heparin vs. geometric mean IC80 fold change of VRC07- 523LS variants.

# Figure S4

**A**

**B**

**C**

**D**

**Antibody**

**Half-life (day) in hFcRn mice (actual)**

**Half-life (day) in humans (model)**

**model b**

**model beta**

**180**

**day µg/mL**

**360**

**day µg/mL**

**180 day fold increase relative to VRC07-523LS (model)**

**360 day fold increase relative to VRC07-523LS (model)**

| VRC07-523LS | 5.4 | 30.8 | 54.28 | 0.022512132 | 1.16 | 0.02 |  | |
| --- | --- | --- | --- | --- | --- | --- | --- | --- |
| VRC07-523LS.v1 | 6.4 | 36.3 | 54.28 | 0.019103644 | 2.15 | 0.07 | 1.8 | 3.4 |
| VRC07-523LS.v11 | 8.1 | 45.6 | 54.28 | 0.015193075 | 4.35 | 0.28 | 3.7 | 13.9 |
| VRC07-523LS.v12 | 7.9 | 44.5 | 54.28 | 0.015567994 | 4.06 | 0.25 | 3.5 | 12.2 |
| VRC07-523LS.v13 | 7.4 | 41.8 | 54.28 | 0.016591568 | 3.38 | 0.17 | 2.9 | 8.4 |
| VRC07-523LS.v14 | 8.4 | 47.3 | 54.28 | 0.014663375 | 4.78 | 0.34 | 4.1 | 16.9 |
| VRC07-523LS.v21 | 9.3 | 52.2 | 54.28 | 0.013274904 | 6.14 | 0.56 | 5.3 | 27.8 |
| VRC07-523LS.v26 | 10.9 | 61.0 | 54.28 | 0.011362219 | 8.67 | 1.12 | 7.4 | 55.4 |
| VRC07-523LS.v32 | 9.3 | 52.2 | 54.28 | 0.013274904 | 6.14 | 0.56 | 5.3 | 27.8 |
| VRC07-523LS.v34 | 10.8 | 60.5 | 54.28 | 0.011465467 | 8.51 | 1.08 | 7.3 | 53.3 |
|  |  |  |  |  |  |  |  |  |

**Antibody**

**Half-life (day) in hFcRn mice (actual)**

**Half-life (day) in humans (model)**

**model b**

**model beta**

**180**

**day µg/mL**

**360**

**day µg/mL**

**180 day fold increase relative to N6LS (model)**

**360 day fold increase relative to N6LS (model)**

| N6LS | 9.0 | 50.6 | 26.67 | 0.013707559 | 1.90 | 0.16 |  | |
| --- | --- | --- | --- | --- | --- | --- | --- | --- |
| N6LS.C1 | 14.1 | 78.6 | 26.67 | 0.008820464 | 4.58 | 0.94 | 2.4 | 5.8 |
| N6LS.C15 | 14.3 | 79.7 | 26.67 | 0.008698842 | 4.68 | 0.98 | 2.5 | 6.1 |
| N6LS.C30 | 15.3 | 85.2 | 26.67 | 0.008137798 | 5.18 | 1.20 | 2.7 | 7.4 |
| N6LS.C35 | 14.5 | 80.8 | 26.67 | 0.008580529 | 4.78 | 1.02 | 2.5 | 6.3 |
| N6LS.C47 | 14.0 | 78.0 | 26.67 | 0.008882559 | 4.53 | 0.92 | 2.4 | 5.7 |
| N6LS.C49 | 14.5 | 80.8 | 26.67 | 0.008580529 | 4.78 | 1.02 | 2.5 | 6.3 |
| N6LS.C51 | 12.8 | 71.4 | 26.67 | 0.00970219 | 3.91 | 0.68 | 2.1 | 4.2 |
| N6LS.C58 | 14.3 | 79.7 | 26.67 | 0.008698842 | 4.68 | 0.98 | 2.5 | 6.1 |

**Figure S4. Estimation of half-life in humans using human FcRn mice data**. (A) Linear Regression of LS-variant antibodies with 100,000 bootstrap resamples used to estimate 95% c.i. (r2 = 0.872) was used to estimate half-life in humans and parameter β in two-compartment models. (B) Scaled models of charge N6LS variants (C) Scaled models of VRC07-523LS charge variants. (D) Table summarizing model parameters and concentration values at 180 and 360 days for the charge variants relative to the parent antibody.

Supplementary Figure 4 Alt Text: Estimation of half-life in humans using human FcRn mice data. Panel a. Linear Regression of LS-variant antibodies with 100,000 bootstrap resamples used to estimate 95% c.i. (r2 = 0.872) was used to estimate half-life in humans and parameter β in two-compartment models. Panel b. Scaled models of charge N6LS variants. Panel c. Scaled models of VRC07-523LS charge variants. Panel d. Table summarizing model parameters and concentration values at 180 and 360 days for the charge variants relative to the parent antibody.

# Table S1

## Table S1. Summary of anti-cardiolipin ELISA.

| **mAb** | | **OD at 450 nm** | | **GPL units** | | **Interpretation** |
| --- | --- | --- | --- | --- | --- | --- |
| **HC** | **LC** | **100 µg/ml** | **33.3 µg/ml** | **100 µg/ml** | **33.3 µg/ml** |  |
| VRC07-523LS_03FR3_H54F | 3aa_del | 0.0711 | 0.043 | 6.32 | 2.94 | Negative |
| VRC07-523LS_03FR3_H54F | 3aa_del, R24D | 0.0416 | 0.0347 | 2.77 | 1.94 | Negative |
| VRC07-523LS_03FR3_H54F | 3aa_del, R54D | 0.0561 | 0.0362 | 4.52 | 2.12 | Negative |
| VRC07-523LS_03FR3_H54F_R19D | 3aa_del, R24D | 0.0355 | 0.0311 | 2.04 | 1.51 | Negative |
| VRC07-523LS_03FR3_H54F_R23D | 3a_del, R24D | 0.0335 | 0.0311 | 1.80 | 1.51 | Negative |
| VRC07-523LS_03FR3_H54F_R23D | 3aa_del, R54D, | 0.0370 | 0.0320 | 2.22 | 1.62 | Negative |
| VRC07-523LS_03FR3_H54F_R82aD | 3aa_del, R24D | 0.0365 | 0.0348 | 2.16 | 1.95 | Negative |
| VRC07-523LS_03FR3_H54F_R82aD | 3aa_del, R54D | 0.0369 | 0.0335 | 2.21 | 1.80 | Negative |
| VRC07-523LS_03FR3_H54F_R19D_23D | 3aa_del | 0.0450 | 0.0325 | 3.18 | 1.68 | Negative |
| VRC07-523LS_03FR3_H54F_R19D_23D | 3aa_del, R24D | 0.0505 | 0.0336 | 3.84 | 1.81 | Negative |
| VRC07-523LS_03FR3_H54F_R19D_89aD | 3aa_del | 0.0361 | 0.0310 | 2.11 | 1.50 | Negative |
| VRC07-523LS_03FR3_H54F_R19D_89aD | 3aa_del, R24D | 0.0351 | 0.0298 | 1.99 | 1.35 | Negative |
| VRC07-523LS_03FR3_H54F_R23_89aD | 3aa_del | 0.0339 | 0.0299 | 1.85 | 1.36 | Negative |
| VRC07-523LS_03FR3_H54F_R23_89aD | 3aa_del, R24D | 0.0315 | 0.0271 | 1.56 | 1.03 | Negative |
| VRC07-523LS_03FR3_H54F_R23_89aD | 3aa_del, R54D | 0.0395 | 0.0328 | 2.52 | 1.71 | Negative |
| N6_03FR3, R19D | LC | 0.1646 | 0.0703 | 7.41 | -2.84 | Negative |
| N6_03FR3, R82aD | LC | 0.0447 | 0.0386 | -5.62 | -6.28 | Negative |
| N6_03FR3 | LC-R18D | 0.0527 | 0.0394 | -4.75 | -6.20 | Negative |
| N6LS | | 0.0733 | 0.0446 | -2.51 | -5.63 | Negative |
| N6LS-03FR3 | | 0.0547 | 0.0394 | -4.53 | -6.20 | Negative |
| 4E10 | | 1.5867 | 1.7065 | 188.73 | 203.15 | High positive |
| VRC07-523LS | | 0.1449 | 0.1016 | 15.21 | 9.99 | Negative |
| VRC07-G54W | | 0.5532 | 0.2679 | 64.34 | 30.01 | Medium positive |

#### GPL score Reactivity

<15 Not reactive

15-20 Indeterminate

>80

20-80

Low to medium positive High positive

Supplementary Table 1 Alt Text: Table shows the summary of Anti-Cardiolipin ELISA.

# Table S2

## Table S2. Neutralizing potency and breadth of VRC07-523LS and N6LS variants on a 12-strain panel.

| <0.001 | .001-.01 | .01-.100 | .100-1.00 | 1.00-10.0 | >10.0 |
| --- | --- | --- | --- | --- | --- |

| Virus | Clade | VRC07- 523LS.v1 | LC- R24D  +R54D | LC-R24D  +R66D | LC-R54D  + R66D | HC-R19D | HC-R23D | HC-R82aD | HC-R19D  +R23D/ LC- R24D+R66 D | HC-R89aD/ LC-R24D+ R66D |
| --- | --- | --- | --- | --- | --- | --- | --- | --- | --- | --- |
| **Q23.17.SG3** | **A** | 0.007 | 0.012 | 0.010 | 0.016 | 0.009 | 0.009 | 0.013 | 0.020 | 0.019 |
| **UG037.8.SG3** | **A** | 0.007 | 0.012 | 0.009 | 0.016 | 0.010 | 0.011 | 0.014 | 0.019 | 0.021 |
| **242-14.SG3** | **AG** | 0.034 | 1.890 | >40 | >40 | 0.056 | 0.061 | 0.070 | 21.700 | >40 |
| **7165.18.SG3** | **B** | 0.616 | 1.480 | 1.410 | 2.670 | 1.860 | 1.070 | 2.030 | 5.660 | 4.230 |
| **AC10.29.SG3** | **B** | 0.178 | 0.370 | 0.304 | 0.467 | 0.424 | 0.307 | 0.311 | 0.955 | 0.453 |
| **JRFL.JB.SG3** | **B** | 0.003 | 0.003 | 0.005 | 0.002 | 0.004 | 0.003 | 0.004 | 0.007 | 0.009 |
| **QH0692.42.SG3** | **B** | 0.033 | 0.072 | 0.061 | 0.095 | 0.101 | 0.058 | 0.081 | 0.176 | 0.162 |
| **DU151.02.SG3** | **C** | 0.017 | 0.014 | >40 | >40 | 0.025 | 0.030 | 0.030 | 25.400 | >40 |
| **DU172.17.SG3** | **C** | 0.043 | 0.109 | 23.900 | >40 | 0.057 | 0.035 | 0.049 | 0.441 | >40 |
| **TV1.29.SG3** | **C** | 0.176 | >40 | >40 | >40 | 0.323 | 0.258 | 0.390 | >40 | >40 |
| **ZM53.12.SG3** | **C** | 0.275 | 0.711 | 0.421 | 0.751 | 0.693 | 0.684 | 0.724 | 2.370 | 1.110 |
| **57128.vrc15.SG3** | **D** | 0.966 | 2.930 | >40 | 40.000 | 1.250 | 0.825 | 1.130 | 36.200 | >40 |

| Median IC50 | 0.039 | 0.109 | 0.183 | 0.281 | 0.079 | 0.060 | 0.076 | 0.955 | 0.162 |
| --- | --- | --- | --- | --- | --- | --- | --- | --- | --- |
| Geometric mean | 0.054 | 0.124 | 0.136 | 0.192 | 0.096 | 0.078 | 0.102 | 0.765 | 0.143 |

| Virus | Clade | VRC07- 523LS.v1 | LC R24D | LC R54D | HC- R19DLC_ R24D | HC_R23D LC_R24D | HC_R23D LC_R54D | HC_ R89aD LC_R24D | HC_ R89aD LC_R54D | HC_R19D  + R23D | HC_R19D  + R23D LC_R24D | HC_R19D  + R89aD | HC_R19D  + R89aD LC_R24D | HC_R23D  + R89aD | HC_R23D  + R89aD LC_R24D | HC_R23D  + R89aD LC_R54D |
| --- | --- | --- | --- | --- | --- | --- | --- | --- | --- | --- | --- | --- | --- | --- | --- | --- |
| Q23.17.SG3 | A | 0.011 | 0.009 | 0.008 | 0.006 | 0.006 | 0.014 | 0.012 | 0.009 | 0.008 | 0.009 | 0.021 | 0.015 | 0.007 | 0.008 | 0.013 |
| UG037.8.SG3 | A | 0.011 | 0.009 | 0.011 | 0.016 | 0.020 | 0.011 | 0.011 | 0.010 | 0.016 | 0.025 | 0.020 | 0.020 | 0.018 | 0.028 | 0.057 |
| 242-14.SG3 | AG | 0.033 | 0.042 | 0.133 | 0.100 | 0.101 | 0.213 | 0.161 | 0.780 | 0.090 | 0.218 | 0.136 | 0.271 | 0.076 | 0.157 | 1.560 |
| 7165.18.SG3 | B | 1.200 | 1.260 | 2.220 | 3.990 | 2.030 | 3.120 | 4.000 | 5.750 | 4.730 | 6.610 | 6.110 | 9.860 | 4.620 | 4.980 | 9.450 |
| AC10.29.SG3 | B | 0.276 | 0.272 | 0.326 | 0.630 | 0.487 | 0.746 | 0.423 | 0.420 | 0.946 | 1.280 | 0.940 | 0.954 | 0.613 | 0.882 | 1.240 |
| JRFL.JB.SG3 | B | 0.004 | 0.005 | 0.006 | 0.008 | 0.004 | 0.007 | 0.009 | 0.012 | 0.008 | 0.010 | 0.011 | 0.012 | 0.005 | 0.007 | 0.012 |
| QH0692.42.SG3 | B | 0.110 | 0.130 | 0.128 | 0.216 | 0.116 | 0.134 | 0.193 | 0.246 | 0.201 | 0.257 | 0.320 | 0.416 | 0.181 | 0.195 | 0.308 |
| DU151.02.SG3 | C | 0.038 | 0.035 | 0.042 | 0.055 | 0.068 | 0.080 | 0.067 | 0.070 | 0.092 | 0.124 | 0.068 | 0.100 | 0.060 | 0.067 | 0.153 |
| DU172.17.SG3 | C | 0.063 | 0.083 | 0.077 | 0.106 | 0.114 | 0.154 | 0.211 | 0.264 | 0.141 | 0.173 | 0.151 | 0.300 | 0.102 | 0.154 | 0.465 |
| TV1.29.SG3 | C | 0.293 | 0.711 | 3.330 | 0.736 | 0.618 | 2.220 | 1.560 | 9.560 | 0.360 | 0.713 | 0.744 | 2.050 | 0.655 | 1.220 | 7.690 |
| ZM53.12.SG3 | C | 0.507 | 0.496 | 0.489 | 0.797 | 1.410 | 1.480 | 1.390 | 0.938 | 1.710 | 3.680 | 1.740 | 2.220 | 0.918 | 1.400 | 2.910 |
| 57128.vrc15.SG3 | D | 1.220 | 1.120 | 1.250 | 0.983 | 1.520 | 4.130 | 2.440 | 2.060 | 0.834 | 2.520 | 3.080 | 3.950 | 2.180 | 1.480 | 4.780 |
|  | | | | | | | | | | | | | | | | |
| Median IC50 | | 0.087 | 0.107 | 0.131 | 0.161 | 0.115 | 0.184 | 0.202 | 0.342 | 0.171 | 0.238 | 0.236 | 0.358 | 0.142 | 0.176 | 0.853 |
| Geometric mean | | 0.089 | 0.099 | 0.137 | 0.151 | 0.140 | 0.219 | 0.202 | 0.273 | 0.169 | 0.270 | 0.245 | 0.336 | 0.155 | 0.205 | 0.519 |

| Virus | Clade |
| --- | --- |
| **Q23.17.SG3** | **A** |
| **UG037.8.SG3** | **A** |
| **242-14.SG3** | **AG** |
| **7165.18.SG3** | **B** |
| **AC10.29.SG3** | **B** |
| **JRFL.JB.SG3** | **B** |
| **QH0692.42.SG3** | **B** |
| **DU151.02.SG3** | **C** |
| **DU172.17.SG3** | **C** |
| **TV1.29.SG3** | **C** |
| **ZM53.12.SG3** | **C** |
| **57128.vrc15.SG3** | **D** |

| N6LS  _03FR3 | HC R89aD | LC_R18D | HC_89aD LC_R18D |
| --- | --- | --- | --- |
| 0.024 | 0.047 | 0.030 | 0.062 |
| 0.019 | 0.035 | 0.020 | 0.040 |
| 5.750 | 30.800 | 7.780 | >40 |
| 3.240 | 11.000 | 3.330 | 11.500 |
| 0.518 | 0.709 | 0.540 | 0.706 |
| 0.008 | 0.020 | 0.011 | 0.022 |
| 0.227 | 0.238 | 0.136 | 0.269 |
| 0.049 | 0.091 | 0.060 | 0.112 |
| 0.048 | 0.107 | 0.084 | 0.117 |
| >40 | >40 | >40 | >40 |
| 0.731 | 1.110 | 0.505 | 1.250 |
| 2.750 | 3.960 | 2.290 | 3.740 |

Geometric mean

Median IC50

| 0.227 | 0.238 | 0.136 | 0.193 |
| --- | --- | --- | --- |
| 0.211 | 0.424 | 0.225 | 0.306 |

IC50 (µg/ml)

Supplementary Table 2 Alt Text: Table shows neutralization breadth and potency of VRC07- 523LS and N6LS variants on a 12-isolate panel.

# Table S3

1. Neutralization potency and heparin chromatography

of VRC07-523LS variants

1. Neutralization potency and heparin chromatography

of N6LS variants

| Iterative optimizations | Variant | Substitution | | Retention volume | IC80 |
| --- | --- | --- | --- | --- | --- |
|  |  | HC | LC | (ml) | Fold change |
| 1st round-Screening of | N6LS.C1 | R82aD | R18D | 6.05 | 0.44 |
| variants incorporating Arg  to Asp substitutions selected based on ASA and an N6-bound gp120 |  |  |  |  | 0.50 |
|  | N6LS.C2 | R82aD | None | 6.13  6.15 |  |
|  | N6LS.C3 | R19D |  |  | 0.76 |
|  | N6LS.C4 | R1D |  | 6.30 | 0.92 |
|  | N6LS.C5 | 03FR3 | R18D | 6.46 | 0.94 |
| structure |  |  |  |  |  |
|  | N6LS.C11 |  | R18E | 6.38 | 0.89 |
|  | N6LS.C12 |  | R42E | 6.29 | 0.84 |
|  | N6LS.C13 | 03FR3 | K45E | 6.22 | 0.79 |
|  | N6LS.C14 |  | R42E, K45E | 6.50 | 0.72 |
| 2nd round- Screening of more variants incorporating Glu substitutions in the heavy and light chains | N6LS.C15 |  | R18E, R42E, K45E | 6.16  6.15 | 0.83 |
|  | N6LS.C16 |  | R42E, K45E, K107E |  | 0.71 |
|  | N6LS.C21 | R19E, R82aE | R18E | 6.04 | 0.21  0.10  0.16 |
|  | N6LS.C22 |  | R42E, K45E | 5.91 |  |
|  | N6LS.C23 |  | R18E, R42E, K45E | 5.82  5.83 |  |
|  | N6LS.C24 |  | R42E, K45E, K107E |  | 0.14 |
|  | N6LS.C25 |  | R18E | 5.88 | 0.13 |
|  | N6LS.C26 | R13E, R19E, R82aE | R42E, K45E | 5.82  5.79 | 0.10  0.08 |
|  | N6LS.C27 |  | R18E, R42E, K45E |  |  |
|  | N6LS.C28 |  | R42E, K45E, K107E | 5.78 | 0.10 |
|  | N6LS.C29 | 03FR3 |  | 6.56 | 0.90 |
|  | N6LS.C30 | K13E |  | 6.03 | 0.55 |
| 3rd round- Screening | N6LS.C31 | R19E | R18E | 6.07 | 0.38 |
|  | N6LS.C32 | R43E |  | 6.37 | 0.77 |
| and optimization of |  |  |  |  |  |
|  | N6LS.C33 | R82aE |  | 6.11 | 0.40 |
| variants incorporating |  |  |  |  |  |
| Arg or Lys to Glu | N6LS.C34 | 03FR3 |  | 6.34 | 0.59 |
| substitutions in the  heavy chain. | N6LS.C35 | K13E | R18E, R42E, K45E | 5.79 | 0.33  0.27 |
|  | N6LS.C36 | R19E |  | 5.93 |  |
|  | N6LS.C37 | R43E |  | 6.17 | 0.46 |
|  | N6LS.C38 | R82aE |  | 5.81 | 0.30 |
|  | N6LS.C39 | R43S | R18E | 6.27 | 0.61 |
|  | N6LS.C40 | R43E |  | 6.22 | 0.62 |
|  | N6LS.C41 | R43S | R18S | 6.30 | 0.72 |
|  | N6LS.C42 | R43E |  | 6.23 | 0.74 |
|  | N6LS.C43 | 03FR3 | R18S, R42S, K45S | 6.27 | 0.71 |
|  | N6LS.C44 | R43S |  | 6.15 | 0.56 |
|  | N6LS.C45 |  | R18D | 6.39 | 1.08 |
|  | N6LS.C46 (11) | 03FR3 | R18E | 6.30  6.25 | 1.14  1.13 |
|  | N6LS.C47 |  | R18Q |  |  |
| Comparison of variants incoporating Arg or Lys to Asp, Gln, Glu, or Ser substitutions | N6LS.C48 |  | R18S | 6.24 | 1.00 |
|  | N6LS.C49 | R82aQ | R18Q | 6.02  6.01 | 0.72  0.72 |
|  | N6LS.C50 | R82aS | R18S |  |  |
|  | N6LS.C51 | K13Q | K18Q | 5.96  5.95 | 0.76  0.73 |
|  | N6LS.C52 |  | K18S |  |  |
|  | N6LS.C53 | K13S | K18Q | 5.96 | 0.79 |
|  | N6LS.C54 |  | K18S | 5.99 | 0.83 |
|  | N6LS.C55 | 03FR3 |  | 6.03 | 0.82 |
|  | N6LS.C56 | K13Q | R18Q, R42Q, K45Q | 5.79 | 0.57 |
|  | N6LS.C57 | K13S |  | 5.80 | 0.64 |
|  | N6LS.C58 |  | K18Q | 6.10 | 0.95 |
|  | N6LS.C59 | K43Q | K18S | 6.09 | 0.94 |
|  | N6LS.C60 |  | R18Q, R42Q, K45Q | 5.89 | 0.62 |
|  | N6LS_03FR3 | 03FR3 | None | 6.65 | 1.00 |

| Iterative optimizations | Variant | Substitution | | Retention time | IC80 |
| --- | --- | --- | --- | --- | --- |
|  |  | HC | LC | (ml) | Fold change |
| 1st round-Screening of variants incorporating Arg to Asp substitutions selected based on ASA and a VRC07-  bound gp120 structure | VRC07-523LS.v1 | None | None | 5.87 | 1.00 |
|  | VRC07-523LS.v11 | None | R24D | 5.73 | 0.83 |
|  | VRC07-523LS.v12 | R19D | None | 5.41  5.41  5.45 | 0.50 |
|  | VRC07-523LS.v13 | R23D, R82aD |  |  | 0.45 |
|  | VRC07-523LS.v14 |  | R24D |  | 0.69 |
| 2nd round-Screening of variants incorporating double or triple substitutions in the heavy chain | VRC07-523LS.v15 | R23D, K13E | R24D | 5.34 | 0.50 |
|  | VRC07-523LS.v16 | R23D, K43E |  | 4.91 | 0.35 |
|  | VRC07-523LS.v17 | R23D, K96E |  | 5.36 | 0.71 |
|  | VRC07-523LS.v18 | K13E, R23D, K96E |  | 5.23 | 0.58 |
|  | VRC07-523LS.v19 | R23D, K13E | R24E | 5.32 | 0.47 |
|  | VRC07-523LS.v20 | R23D, K43E |  | 4.89 | 0.34 |
|  | VRC07-523LS.v21 | R23D, K96E |  | 5.34 | 0.66 |
|  | VRC07-523LS.v22 | K13E, R23D, K96E |  | 5.23 | 0.52 |
| 3rd round- Screening of variants incorporating Arg or Lys to Gln, Glu, or Ser sustitutions | VRC07-523LS.v23 | None | R24S | 5.83 | 0.91  0.90 |
|  | VRC07-523LS.v24 |  | R24Q | 5.69  5.75 |  |
|  | VRC07-523LS.v25 |  | R24E |  | 0.83 |
|  | VRC07-523LS.v26 | R23S | R24S | 5.72  5.68 | 1.04 |
|  | VRC07-523LS.v27 |  | R24Q |  | 0.89  0.85 |
|  | VRC07-523LS.v28 |  | R24E | 5.57 |  |
|  | VRC07-523LS.v29 | R23Q | R24S | 5.63  5.64 | 0.91 |
|  | VRC07-523LS.v30 |  | R24Q |  | 0.83  0.81 |
|  | VRC07-523LS.v31 |  | R24E | 5.53 |  |
|  | VRC07-523LS.v32 | R23S, K96S | R24S | 5.45  5.49  5.38 | 0.84 |
|  | VRC07-523LS.v33 |  | R24Q |  | 0.66 |
|  | VRC07-523LS.v34 |  | R24E |  | 0.71 |
|  | VRC07-523LS.v35 | R23Q, K96Q | R24S | 5.45 | 0.72 |
|  | VRC07-523LS.v36 |  | R24Q | 5.49 | 0.79 |
|  | VRC07-523LS.v37 |  | R24E | 5.42 | 0.66 |

All heavy chains contained the G54F and 03FR3 loop mutations. All light chains contained the 3aa_del mutation.

Favorable Unfavorable

All heavy chains contained the 03FR3 loop mutation.

Supplementary Table 3 Alt Text: Table 3a. Table shows neutralization potency and heparin chromatography of VRC07-523LS variants. Table 3b. Table shows neutralization potency and heparin chromatography of N6LS variants.

# Table S4

## Table S4. Comparison of protein expression between VRC07-523LS variants with light chain R24D and R24E mutation.

| Variant | HC | LC | Yield (mg/50 ml) | Heparin | Geomean (µg/ml) | |
| --- | --- | --- | --- | --- | --- | --- |
|  |  |  |  | RV (ml) | IC50 | IC80 |
| VRC07-523LS.v15 | R23D/K13E | R24D | 7.6 | 5 | 0.133 | 0.440 |
| VRC07-523LS.v16 | R23D/K43E |  | 0.8  1.4  1.8 | 4.6 | 0.184 | 0.622 |
| VRC07-523LS.v17 | R23D/K96E |  |  | 5.02 | 0.095 | 0.307 |
| VRC07-523LS.v18 | R23D/K13E/K96E |  |  | 4.9 | 0.122 | 0.382 |
| VRC07-523LS.v19 | R23D/K13E | R24E | 4.5 | 4.99 | 0.175 | 0.472 |
| VRC07-523LS.v20 | R23D/K43E |  | 9.5 | 4.58 | 0.223 | 0.645 |
| VRC07-523LS.v21 | R23D/K96E |  | 7.2 | 5 | 0.119 | 0.333 |
| VRC07-523LS.v22 | R23D/K13E/K96E |  | 9.2 | 4.9 | 0.156 | 0.422 |

Favorable Unfavorable

Supplementary Table 4 Alt Text: Tables shows comparison of protein yields between VRC07- 523LS variants with light chain R24D and R24E mutation.

# Table S5

## TableS5. Relative frequency of amino acids in the variable domain of human antibodies.

Relative frequency of amino acids in HIV-1 antibodies

| **Kabat Position** | **A** | **C** | **D** | **E** | **F** | **G** | **H** | **I** | **K** | **L** | **M** | **N** | **P** | **Q** | **R** | **S** | **T** | **V** | **W** | **Y** | **K + R** |
| --- | --- | --- | --- | --- | --- | --- | --- | --- | --- | --- | --- | --- | --- | --- | --- | --- | --- | --- | --- | --- | --- |
| H:12 | 0.68% | 0.00% | 0.02% | 0.11% | 0.11% | 0.20% | 0.00% | 1.24% | 34.15% | 5.39% | 0.23% | 0.02% | 0.00% | 0.42% | 4.31% | 0.09% | 0.17% | 45.86% | 0.02% | 0.00% | 38.46% |
| H:13 | 0.11% | 0.00% | 0.05% | 1.71% | 0.00% | 0.15% | 0.17% | 0.05% | 58.63% | 0.21% | 0.12% | 0.33% | 0.23% | 23.30% | 4.43% | 0.50% | 3.05% | 0.00% | 0.00% | 0.00% | 63.06% |
| H:19 | 0.08% | 0.00% | 0.00% | 0.11% | 0.15% | 0.11% | 0.00% | 0.03% | 24.60% | 0.02% | 0.00% | 0.27% | 0.06% | 0.12% | 38.53% | 27.38% | 8.41% | 0.00% | 0.12% | 0.00% | 63.14% |
| H:23 | 29.88% | 0.00% | 1.32% | 3.67% | 0.05% | 3.20% | 0.00% | 0.44% | 20.88% | 0.18% | 0.06% | 0.82% | 0.02% | 2.78% | 6.06% | 6.59% | 17.41% | 6.62% | 0.00% | 0.02% | 26.94% |
| H:38 | 0.00% | 0.00% | 0.00% | 0.00% | 0.00% | 0.03% | 0.02% | 0.00% | 0.00% | 0.02% | 0.00% | 0.00% | 0.00% | 0.26% | 99.64% | 0.02% | 0.00% | 0.00% | 0.02% | 0.00% | 99.64% |
| H:43 | 0.03% | 0.00% | 0.00% | 0.80% | 0.05% | 1.09% | 0.71% | 0.00% | 66.02% | 0.03% | 0.21% | 0.14% | 0.00% | 18.36% | 11.31% | 0.06% | 0.38% | 0.00% | 0.03% | 0.73% | 77.33% |
| H:52B | 0.03% | 0.03% | 0.09% | 0.38% | 0.03% | 0.18% | 0.00% | 0.09% | 1.67% | 0.03% | 0.00% | 0.20% | 0.12% | 0.06% | 0.71% | 0.24% | 0.50% | 0.00% | 0.00% | 0.06% | 2.38% |
| H:62 | 2.14% | 0.02% | 0.15% | 0.30% | 0.47% | 0.27% | 0.20% | 0.30% | 14.90% | 0.09% | 0.15% | 3.04% | 2.49% | 8.71% | 6.63% | 58.79% | 1.09% | 0.08% | 0.00% | 0.12% | 21.54% |
| H:64 | 0.18% | 0.00% | 0.05% | 3.16% | 0.00% | 0.23% | 0.24% | 0.03% | 49.46% | 0.17% | 0.62% | 0.56% | 0.65% | 30.95% | 12.22% | 0.32% | 0.53% | 0.00% | 0.06% | 0.53% | 61.68% |
| H:66 | 0.02% | 0.00% | 0.00% | 0.00% | 0.00% | 0.06% | 0.87% | 0.02% | 3.41% | 0.08% | 0.00% | 0.00% | 0.09% | 2.16% | 92.85% | 0.06% | 0.02% | 0.02% | 0.32% | 0.00% | 96.27% |
| H:71 | 9.14% | 0.00% | 0.05% | 0.55% | 0.12% | 0.44% | 0.02% | 2.94% | 1.52% | 2.99% | 0.52% | 0.05% | 0.36% | 0.08% | 56.53% | 0.68% | 3.04% | 20.88% | 0.08% | 0.02% | 58.05% |
| H:75 | 1.85% | 0.00% | 0.35% | 6.34% | 0.02% | 0.26% | 0.08% | 9.56% | 45.96% | 0.67% | 3.02% | 2.05% | 0.05% | 5.42% | 7.18% | 3.23% | 12.92% | 0.74% | 0.12% | 0.03% | 53.13% |
| H:82A | 0.52% | 0.00% | 2.09% | 0.03% | 0.06% | 0.83% | 0.42% | 0.80% | 3.29% | 0.08% | 0.15% | 29.25% | 0.12% | 0.09% | 21.82% | 29.32% | 10.55% | 0.21% | 0.08% | 0.27% | 25.12% |
| H:94 | 1.62% | 0.32% | 0.00% | 0.05% | 0.20% | 1.21% | 0.87% | 0.47% | 9.82% | 0.77% | 2.81% | 0.33% | 0.03% | 0.12% | 68.48% | 7.76% | 4.58% | 0.27% | 0.03% | 0.26% | 78.30% |
| H:96 | 6.24% | 0.15% | 4.96% | 2.46% | 1.76% | 18.05% | 1.58% | 2.03% | 4.80% | 5.83% | 1.09% | 1.94% | 14.33% | 2.16% | 13.70% | 6.97% | 2.96% | 3.84% | 1.76% | 2.97% | 18.50% |
|  |  |  |  |  |  |  |  |  |  |  |  |  |  |  |  |  |  |  |  |  |  |
| L:18 | 0.34% | 0.00% | 0.03% | 0.20% | 0.00% | 0.83% | 0.00% | 0.06% | 22.80% | 0.01% | 0.37% | 0.62% | 4.18% | 0.11% | 44.25% | 10.27% | 15.78% | 0.08% | 0.06% | 0.00% | 67.06% |
| L:24 | 0.80% | 0.01% | 0.01% | 0.46% | 0.13% | 3.69% | 0.00% | 0.14% | 6.48% | 0.03% | 0.03% | 0.18% | 0.03% | 1.88% | 38.42% | 33.96% | 13.32% | 0.13% | 0.21% | 0.08% | 44.90% |
| L:39 | 0.21% | 0.01% | 0.00% | 0.14% | 3.07% | 0.00% | 6.36% | 0.84% | 46.62% | 25.71% | 0.03% | 0.28% | 0.32% | 0.93% | 11.37% | 0.13% | 0.94% | 2.31% | 0.00% | 0.72% | 57.98% |
| L:42 | 1.98% | 0.00% | 0.03% | 0.46% | 0.00% | 0.55% | 0.52% | 0.06% | 19.40% | 1.00% | 0.11% | 0.31% | 0.01% | 42.57% | 2.26% | 1.72% | 28.91% | 0.10% | 0.00% | 0.00% | 21.66% |
| L:45 | 0.17% | 0.00% | 0.15% | 2.10% | 0.00% | 0.15% | 0.13% | 0.63% | 52.90% | 0.53% | 0.25% | 1.51% | 0.01% | 3.49% | 29.33% | 0.55% | 2.32% | 5.75% | 0.00% | 0.01% | 82.23% |
| L:54 | 0.01% | 0.00% | 0.00% | 0.01% | 0.03% | 0.17% | 0.00% | 0.00% | 1.25% | 14.25% | 0.00% | 0.01% | 0.07% | 0.10% | 83.33% | 0.20% | 0.10% | 0.28% | 0.15% | 0.00% | 84.58% |
| L:66 | 0.80% | 0.00% | 0.13% | 0.25% | 0.00% | 44.30% | 0.03% | 1.88% | 38.09% | 0.49% | 0.10% | 4.97% | 0.07% | 0.46% | 3.00% | 2.69% | 1.06% | 1.58% | 0.08% | 0.01% | 41.09% |
| L:103 | 0.73% | 0.00% | 0.01% | 0.68% | 0.01% | 0.01% | 0.11% | 0.07% | 82.67% | 0.03% | 0.17% | 0.98% | 0.00% | 1.10% | 9.24% | 0.13% | 2.28% | 0.00% | 0.01% | 0.00% | 91.91% |
| L:107 | 0.00% | 0.00% | 0.00% | 0.14% | 0.00% | 0.00% | 0.00% | 0.24% | 36.59% | 50.67% | 0.00% | 0.00% | 0.03% | 0.27% | 1.10% | 0.00% | 0.15% | 0.14% | 0.00% | 0.00% | 37.68% |

abYsis server at <http://www.abysis.org/abysis/searches/distributions/distributions.cgi> High Low

Supplementary Table 5 Alt Text: Table shows relative frequency of amino acids in the variable domain of human antibodies.

# Table S6

**Table S6. Summary of Intrinsic Properties Risk Assessment.**

Supplementary Table 6 Alt Text: Table shows manufacturability and biophysical risk assessment.

# Table S7

## Table S7. Amino acid sequences of VRC07-523LS and N6LS variants.

#### >VRC07-523LS.v11 HC

QVRLSQSGGQMKKPGDSMRISCRASGYEFINCPINWIRLAPGKRPEWMGWMKPRFGAVSYARQLQGRVTMTRQLSQDPDDPDWGTAFLELRSLTSDDTAVYFCTRGKYCTARDYYNWDFEHWGQGTPVTVSSASTKGPSVFPLAPSSKSTSGGTAALGCLVKDYFPE PVTVSWNSGALTSGVHTFPAVLQSSGLYSLSSVVTVPSSSLGTQTYICNVNHKPSNTKVDKKVEPKSCDKTHTCPPCPAPELLGGPSVFLFPPKPKDTLMISRTPEVTCVVVDVSHEDPEVKFNWYVDGVEVHNAKTKPREEQYNSTYRVVSVLTVLHQDWLNGKEY KCKVSNKALPAPIEKTISKAKGQPREPQVYTLPPSRDELTKNQVSLTCLVKGFYPSDIAVEWESNGQPENNYKTTPPVLDSDGSFFLYSKLTVDKSRWQQGNVFSCSVLHEALHSHYTQKSLSLSPGK

#### >VRC07-523LS.v11 LC

LTQSPGTLSLSPGETAIISC**D**TSQYGSLAWYQQRPGQAPRLVIYSGSTRAAGIPDRFSGSRWGPDYNLTISNLESGDFGVYYCQQYEFFGQGTKVQVDIKRTVAAPSVFIFPPSDEQLKSGTASVVCLLNNFYPREAKVQWKVDNALQSGNSQESVTEQDSKDSTYS LSSTLTLSKADYEKHKVYACEVTHQGLSSPVTKSFNRGEC

#### >VRC07-523LS.v12 HC

QVRLSQSGGQMKKPGDSM**D**ISCRASGYEFINCPINWIRLAPGKRPEWMGWMKPRFGAVSYARQLQGRVTMTRQLSQDPDDPDWGTAFLELRSLTSDDTAVYFCTRGKYCTARDYYNWDFEHWGQGTPVTVSSASTKGPSVFPLAPSSKSTSGGTAALGCLVKDYFPE PVTVSWNSGALTSGVHTFPAVLQSSGLYSLSSVVTVPSSSLGTQTYICNVNHKPSNTKVDKKVEPKSCDKTHTCPPCPAPELLGGPSVFLFPPKPKDTLMISRTPEVTCVVVDVSHEDPEVKFNWYVDGVEVHNAKTKPREEQYNSTYRVVSVLTVLHQDWLNGKEY KCKVSNKALPAPIEKTISKAKGQPREPQVYTLPPSRDELTKNQVSLTCLVKGFYPSDIAVEWESNGQPENNYKTTPPVLDSDGSFFLYSKLTVDKSRWQQGNVFSCSVLHEALHSHYTQKSLSLSPGK

#### >VRC07-523LS.v12 LC

LTQSPGTLSLSPGETAIISC**R**TSQYGSLAWYQQRPGQAPRLVIYSGSTRAAGIPDRFSGSRWGPDYNLTISNLESGDFGVYYCQQYEFFGQGTKVQVDIKRTVAAPSVFIFPPSDEQLKSGTASVVCLLNNFYPREAKVQWKVDNALQSGNSQESVTEQDSKDSTYS LSSTLTLSKADYEKHKVYACEVTHQGLSSPVTKSFNRGEC

#### >VRC07-523LS.v13 HC

QVRLSQSGGQMKKPGDSM**D**ISCRASGYEFINCPINWIRLAPGKRPEWMGWMKPRFGAVSYARQLQGRVTMTRQLSQDPDDPDWGTAFLELDSLTSDDTAVYFCTRGKYCTARDYYNWDFEHWGQGTPVTVSSASTKGPSVFPLAPSSKSTSGGTAALGCLVKDYFPE PVTVSWNSGALTSGVHTFPAVLQSSGLYSLSSVVTVPSSSLGTQTYICNVNHKPSNTKVDKKVEPKSCDKTHTCPPCPAPELLGGPSVFLFPPKPKDTLMISRTPEVTCVVVDVSHEDPEVKFNWYVDGVEVHNAKTKPREEQYNSTYRVVSVLTVLHQDWLNGKEY KCKVSNKALPAPIEKTISKAKGQPREPQVYTLPPSRDELTKNQVSLTCLVKGFYPSDIAVEWESNGQPENNYKTTPPVLDSDGSFFLYSKLTVDKSRWQQGNVFSCSVLHEALHSHYTQKSLSLSPGK

#### >VRC07-523LS.v13 LC

LTQSPGTLSLSPGETAIISC**R**TSQYGSLAWYQQRPGQAPRLVIYSGSTRAAGIPDRFSGSRWGPDYNLTISNLESGDFGVYYCQQYEFFGQGTKVQVDIKRTVAAPSVFIFPPSDEQLKSGTASVVCLLNNFYPREAKVQWKVDNALQSGNSQESVTEQDSKDSTYS LSSTLTLSKADYEKHKVYACEVTHQGLSSPVTKSFNRGEC

#### >VRC07-523LS.v14 HC

QVRLSQSGGQMKKPGDSMRISCDASGYEFINCPINWIRLAPGKRPEWMGWMKPRFGAVSYARQLQGRVTMTRQLSQDPDDPDWGTAFLELRSLTSDDTAVYFCTRGKYCTARDYYNWDFEHWGQGTPVTVSSASTKGPSVFPLAPSSKSTSGGTAALGCLVKDYFPE PVTVSWNSGALTSGVHTFPAVLQSSGLYSLSSVVTVPSSSLGTQTYICNVNHKPSNTKVDKKVEPKSCDKTHTCPPCPAPELLGGPSVFLFPPKPKDTLMISRTPEVTCVVVDVSHEDPEVKFNWYVDGVEVHNAKTKPREEQYNSTYRVVSVLTVLHQDWLNGKEY KCKVSNKALPAPIEKTISKAKGQPREPQVYTLPPSRDELTKNQVSLTCLVKGFYPSDIAVEWESNGQPENNYKTTPPVLDSDGSFFLYSKLTVDKSRWQQGNVFSCSVLHEALHSHYTQKSLSLSPGK

#### >VRC07-523LS.v14 HC

LTQSPGTLSLSPGETAIISC**D**TSQYGSLAWYQQRPGQAPRLVIYSGSTRAAGIPDRFSGSRWGPDYNLTISNLESGDFGVYYCQQYEFFGQGTKVQVDIKRTVAAPSVFIFPPSDEQLKSGTASVVCLLNNFYPREAKVQWKVDNALQSGNSQESVTEQDSKDSTYS LSSTLTLSKADYEKHKVYACEVTHQGLSSPVTKSFNRGEC

#### >VRC07-523LS.v21 HC

QVRLSQSGGQMKKPGDSMRISCDASGYEFINCPINWIRLAPGKRPEWMGWMKPRFGAVSYARQLQGRVTMTRQLSQDPDDPDWGTAFLELRSLTSDDTAVYFCTRGEYCTARDYYNWDFEHWGQGTPVTVSSASTKGPSVFPLAPSSKSTSGGTAALGCLVKDYFPE PVTVSWNSGALTSGVHTFPAVLQSSGLYSLSSVVTVPSSSLGTQTYICNVNHKPSNTKVDKKVEPKSCDKTHTCPPCPAPELLGGPSVFLFPPKPKDTLMISRTPEVTCVVVDVSHEDPEVKFNWYVDGVEVHNAKTKPREEQYNSTYRVVSVLTVLHQDWLNGKEY KCKVSNKALPAPIEKTISKAKGQPREPQVYTLPPSRDELTKNQVSLTCLVKGFYPSDIAVEWESNGQPENNYKTTPPVLDSDGSFFLYSKLTVDKSRWQQGNVFSCSVLHEALHSHYTQKSLSLSPGK

#### >VRC07-523LS.v21 LC

LTQSPGTLSLSPGETAIISC**E**TSQYGSLAWYQQRPGQAPRLVIYSGSTRAAGIPDRFSGSRWGPDYNLTISNLESGDFGVYYCQQYEFFGQGTKVQVDIKRTVAAPSVFIFPPSDEQLKSGTASVVCLLNNFYPREAKVQWKVDNALQSGNSQESVTEQDSKDSTYS LSSTLTLSKADYEKHKVYACEVTHQGLSSPVTKSFNRGEC

#### >VRC07-523LS.v26 HC

QVRLSQSGGQMKKPGDSMRISCSASGYEFINCPINWIRLAPGKRPEWMGWMKPRFGAVSYARQLQGRVTMTRQLSQDPDDPDWGTAFLELRSLTSDDTAVYFCTRGKYCTARDYYNWDFEHWGQGTPVTVSSASTKGPSVFPLAPSSKSTSGGTAALGCLVKDYFPE PVTVSWNSGALTSGVHTFPAVLQSSGLYSLSSVVTVPSSSLGTQTYICNVNHKPSNTKVDKKVEPKSCDKTHTCPPCPAPELLGGPSVFLFPPKPKDTLMISRTPEVTCVVVDVSHEDPEVKFNWYVDGVEVHNAKTKPREEQYNSTYRVVSVLTVLHQDWLNGKEY KCKVSNKALPAPIEKTISKAKGQPREPQVYTLPPSRDELTKNQVSLTCLVKGFYPSDIAVEWESNGQPENNYKTTPPVLDSDGSFFLYSKLTVDKSRWQQGNVFSCSVLHEALHSHYTQKSLSLSPGK

#### >VRC07-523LS.v26 LC

LTQSPGTLSLSPGETAIISC**S**TSQYGSLAWYQQRPGQAPRLVIYSGSTRAAGIPDRFSGSRWGPDYNLTISNLESGDFGVYYCQQYEFFGQGTKVQVDIKRTVAAPSVFIFPPSDEQLKSGTASVVCLLNNFYPREAKVQWKVDNALQSGNSQESVTEQDSKDSTYS LSSTLTLSKADYEKHKVYACEVTHQGLSSPVTKSFNRGEC

#### >VRC07-523LS.v32 HC

QVRLSQSGGQMKKPGDSMRISCSASGYEFINCPINWIRLAPGKRPEWMGWMKPRFGAVSYARQLQGRVTMTRQLSQDPDDPDWGTAFLELRSLTSDDTAVYFCTRGSYCTARDYYNWDFEHWGQGTPVTVSSASTKGPSVFPLAPSSKSTSGGTAALGCLVKDYFPE PVTVSWNSGALTSGVHTFPAVLQSSGLYSLSSVVTVPSSSLGTQTYICNVNHKPSNTKVDKKVEPKSCDKTHTCPPCPAPELLGGPSVFLFPPKPKDTLMISRTPEVTCVVVDVSHEDPEVKFNWYVDGVEVHNAKTKPREEQYNSTYRVVSVLTVLHQDWLNGKEY KCKVSNKALPAPIEKTISKAKGQPREPQVYTLPPSRDELTKNQVSLTCLVKGFYPSDIAVEWESNGQPENNYKTTPPVLDSDGSFFLYSKLTVDKSRWQQGNVFSCSVLHEALHSHYTQKSLSLSPGK

#### >VRC07-523LS.v32 LC

LTQSPGTLSLSPGETAIISC**S**TSQYGSLAWYQQRPGQAPRLVIYSGSTRAAGIPDRFSGSRWGPDYNLTISNLESGDFGVYYCQQYEFFGQGTKVQVDIKRTVAAPSVFIFPPSDEQLKSGTASVVCLLNNFYPREAKVQWKVDNALQSGNSQESVTEQDSKDSTYS LSSTLTLSKADYEKHKVYACEVTHQGLSSPVTKSFNRGEC

#### >VRC07-523LS.v34 HC

QVRLSQSGGQMKKPGDSMRISCSASGYEFINCPINWIRLAPGKRPEWMGWMKPRFGAVSYARQLQGRVTMTRQLSQDPDDPDWGTAFLELRSLTSDDTAVYFCTRGSYCTARDYYNWDFEHWGQGTPVTVSSASTKGPSVFPLAPSSKSTSGGTAALGCLVKDYFPE PVTVSWNSGALTSGVHTFPAVLQSSGLYSLSSVVTVPSSSLGTQTYICNVNHKPSNTKVDKKVEPKSCDKTHTCPPCPAPELLGGPSVFLFPPKPKDTLMISRTPEVTCVVVDVSHEDPEVKFNWYVDGVEVHNAKTKPREEQYNSTYRVVSVLTVLHQDWLNGKEY KCKVSNKALPAPIEKTISKAKGQPREPQVYTLPPSRDELTKNQVSLTCLVKGFYPSDIAVEWESNGQPENNYKTTPPVLDSDGSFFLYSKLTVDKSRWQQGNVFSCSVLHEALHSHYTQKSLSLSPGK

#### >VRC07-523LS.v34 LC

LTQSPGTLSLSPGETAIISC**E**TSQYGSLAWYQQRPGQAPRLVIYSGSTRAAGIPDRFSGSRWGPDYNLTISNLESGDFGVYYCQQYEFFGQGTKVQVDIKRTVAAPSVFIFPPSDEQLKSGTASVVCLLNNFYPREAKVQWKVDNALQSGNSQESVTEQDSKDSTYS LSSTLTLSKADYEKHKVYACEVTHQGLSSPVTKSFNRGEC

#### >N6LS.C1 HC

RAHLVQSGTAMKKPGASVRVSCQTSGYTFTAHILFWFRQAPGRGLEWVGWIKPQYGAVNFGGGFRDRVTLTRQLSQDPDDPDWGIAYMDI**D**GLKPDDTAVYYCARDRSYGDSSWALDAWGQGTTVVVSAASTKGPSVFPLAPSSKSTSGGTAALGCLVKDYFPEPVT VSWNSGALTSGVHTFPAVLQSSGLYSLSSVVTVPSSSLGTQTYICNVNHKPSNTKVDKKVEPKSCDKTHTCPPCPAPELLGGPSVFLFPPKPKDTLMISRTPEVTCVVVDVSHEDPEVKFNWYVDGVEVHNAKTKPREEQYNSTYRVVSVLTVLHQDWLNGKEYKCK VSNKALPAPIEKTISKAKGQPREPQVYTLPPSRDELTKNQVSLTCLVKGFYPSDIAVEWESNGQPENNYKTTPPVLDSDGSFFLYSKLTVDKSRWQQGNVFSCSVLHEALHSHYTQKSLSLSPGK

#### >N6LS.C1 LC

YIHVTQSPSSLSVSIGD**D**VTINCQTSQGVGSDLHWYQHKPGRAPKLLIHHTSSVEDGVPSRFSGSGFHTSFNLTISDLQADDIATYYCQVLQFFGRGSRLHIKRTVAAPSVFIFPPSDEQLKSGTASVVCLLNNFYPREAKVQWKVDNALQSGNSQESVTEQDSKDS TYSLSSTLTLSKADYEKHKVYACEVTHQGLSSPVTKSFNRGEC

#### >N6LS.C15 HC

RAHLVQSGTAMKKPGASVRVSCQTSGYTFTAHILFWFRQAPGRGLEWVGWIKPQYGAVNFGGGFRDRVTLTRQLSQDPDDPDWGIAYMDI**R**GLKPDDTAVYYCARDRSYGDSSWALDAWGQGTTVVVSAASTKGPSVFPLAPSSKSTSGGTAALGCLVKDYFPEPVT VSWNSGALTSGVHTFPAVLQSSGLYSLSSVVTVPSSSLGTQTYICNVNHKPSNTKVDKKVEPKSCDKTHTCPPCPAPELLGGPSVFLFPPKPKDTLMISRTPEVTCVVVDVSHEDPEVKFNWYVDGVEVHNAKTKPREEQYNSTYRVVSVLTVLHQDWLNGKEYKCK VSNKALPAPIEKTISKAKGQPREPQVYTLPPSRDELTKNQVSLTCLVKGFYPSDIAVEWESNGQPENNYKTTPPVLDSDGSFFLYSKLTVDKSRWQQGNVFSCSVLHEALHSHYTQKSLSLSPGK

#### >N6LS.C15 LC

YIHVTQSPSSLSVSIGD**E**VTINCQTSQGVGSDLHWYQHKPG**E**AP**E**LLIHHTSSVEDGVPSRFSGSGFHTSFNLTISDLQADDIATYYCQVLQFFGRGSRLHIKRTVAAPSVFIFPPSDEQLKSGTASVVCLLNNFYPREAKVQWKVDNALQSGNSQESVTEQDSKDS TYSLSSTLTLSKADYEKHKVYACEVTHQGLSSPVTKSFNRGEC

#### >N6LS.C30 HC

RAHLVQSGTAMK**E**PGASVRVSCQTSGYTFTAHILFWFRQAPGRGLEWVGWIKPQYGAVNFGGGFRDRVTLTRQLSQDPDDPDWGIAYMDIRGLKPDDTAVYYCARDRSYGDSSWALDAWGQGTTVVVSAASTKGPSVFPLAPSSKSTSGGTAALGCLVKDYFPEPVT VSWNSGALTSGVHTFPAVLQSSGLYSLSSVVTVPSSSLGTQTYICNVNHKPSNTKVDKKVEPKSCDKTHTCPPCPAPELLGGPSVFLFPPKPKDTLMISRTPEVTCVVVDVSHEDPEVKFNWYVDGVEVHNAKTKPREEQYNSTYRVVSVLTVLHQDWLNGKEYKCK VSNKALPAPIEKTISKAKGQPREPQVYTLPPSRDELTKNQVSLTCLVKGFYPSDIAVEWESNGQPENNYKTTPPVLDSDGSFFLYSKLTVDKSRWQQGNVFSCSVLHEALHSHYTQKSLSLSPGK

#### >N6LS.C30 LC

YIHVTQSPSSLSVSIGD**E**VTINCQTSQGVGSDLHWYQHKPGRAPKLLIHHTSSVEDGVPSRFSGSGFHTSFNLTISDLQADDIATYYCQVLQFFGRGSRLHIKRTVAAPSVFIFPPSDEQLKSGTASVVCLLNNFYPREAKVQWKVDNALQSGNSQESVTEQDSKDS TYSLSSTLTLSKADYEKHKVYACEVTHQGLSSPVTKSFNRGEC

#### >N6LS.C35 HC

RAHLVQSGTAMK**E**PGASVRVSCQTSGYTFTAHILFWFRQAPGRGLEWVGWIKPQYGAVNFGGGFRDRVTLTRQLSQDPDDPDWGIAYMDIRGLKPDDTAVYYCARDRSYGDSSWALDAWGQGTTVVVSAASTKGPSVFPLAPSSKSTSGGTAALGCLVKDYFPEPVT VSWNSGALTSGVHTFPAVLQSSGLYSLSSVVTVPSSSLGTQTYICNVNHKPSNTKVDKKVEPKSCDKTHTCPPCPAPELLGGPSVFLFPPKPKDTLMISRTPEVTCVVVDVSHEDPEVKFNWYVDGVEVHNAKTKPREEQYNSTYRVVSVLTVLHQDWLNGKEYKCK VSNKALPAPIEKTISKAKGQPREPQVYTLPPSRDELTKNQVSLTCLVKGFYPSDIAVEWESNGQPENNYKTTPPVLDSDGSFFLYSKLTVDKSRWQQGNVFSCSVLHEALHSHYTQKSLSLSPGK

#### >N6LS.C35 LC

YIHVTQSPSSLSVSIGD**E**VTINCQTSQGVGSDLHWYQHKPG**E**AP**E**LLIHHTSSVEDGVPSRFSGSGFHTSFNLTISDLQADDIATYYCQVLQFFGRGSRLHIKRTVAAPSVFIFPPSDEQLKSGTASVVCLLNNFYPREAKVQWKVDNALQSGNSQESVTEQDSKDS TYSLSSTLTLSKADYEKHKVYACEVTHQGLSSPVTKSFNRGEC

Supplementary Table 7 Alt Text: Table shows amino acid sequences of VRC07-523LS and N6LS variants.
